# CBX8 suppresses autophagy-dependent senescence in colorectal cancer by modulating the mTOR signaling pathway

**DOI:** 10.7150/ijbs.126032

**Published:** 2026-02-26

**Authors:** Tiankang Li, Enjian Zhang, Xin Liu, Hui Zhou, Pengbo Zhang, Chong Zhang, Xiuzhong Zhang, Nai Wu, Shuai Gong, Zeqiang Ren, Jie Ding, Yi Zhang

**Affiliations:** 1 Department of Gastrointestinal Surgery, Affiliated Hospital of Xuzhou Medical University, Xuzhou, 221000, China.; 2 Institute of Digestive Diseases, Xuzhou Medical University, Xuzhou, 221000, China.; 3 Department of Breast Surgery, Shanghai First Maternity and Infant Hospital, School of Medicine, Tongji University, Shanghai, 200092, China.; 4 Department of Endocrinology, Affiliated Hospital of Xuzhou Medical University, Xuzhou, 221000, China.; 5 Department of Gastrointestinal Surgery, Guizhou Provincial People's Hospital; NHC Key Laboratory of Pulmonary Immunological Diseases, Guizhou Provincial People's Hospital, Guiyang, 550002, China.; 6 Department of Epigenetics and Molecular Carcinogenesis, University of Texas MD Anderson Cancer Center, Houston, TX, 77030, USA.

**Keywords:** colorectal cancer, CBX8, autophagy, senescence, senolytic

## Abstract

Colorectal cancer (CRC) is among the most common cancers worldwide. Cellular senescence, characterized by an irreversible state of growth arrest, has been recognized as a promising therapeutic strategy for combating cancer. Here, the oncogenic role of Chromobox homolog 8 (CBX8) in CRC and its regulatory mechanisms in cell senescence and transcriptional regulation were systematically investigated. We demonstrated that CBX8 deficiency suppresses colorectal tumorigenesis and promotes tumor cell senescence in both *in vivo* and *in vitro* models. Mechanistically, CBX8 inhibits autophagy-dependent senescence in CRC by modulating the mTOR signaling pathway through transcriptional repression of DDIT4, a known negative regulator of mTOR. CBX8 achieves this by recruiting TRIM28 to bind the promoter region of DDIT4, thereby maintaining the H3K27me3 modification status and repressing expression of DDIT4. Furthermore, our findings highlight the therapeutic potential of CBX8 inhibitors in combination with senescence-targeting agents, which significantly enhances antitumor effects in CRC xenograft models. These results provide novel insights into the molecular mechanisms underlying CRC progression and underscore the potential of CBX8 as a therapeutic target for developing targeted therapies and senolytic-based anticancer strategies. This study advances our understanding of CRC pathogenesis and offers promising directions for precision medicine in CRC treatment.

## Introduction

Colorectal cancer (CRC) ranks as the fourth leading cause of cancer-related mortality worldwide, accounting for approximately 9.2% of all cancer deaths 1. The incidence rates of CRC are rapidly increasing due to gradual lifestyle changes.

Cellular senescence is characterized by a stable cell-cycle arrest accompanied by distinct morphological and phenotypic alterations, including increased cell size, accumulation of cytoplasmic vacuoles, and the secretion of a variety of factors collectively termed senescence-associated secretory phenotypes (SASPs) 2. CRC is recognized as a quintessential age-related disease 3; the association between CRC and cellular senescence has become increasingly evident. For instance, inhibition of METTL3 or specifically targeting CDKN2B methylation can suppress CRC senescence, facilitate M2 macrophage polarization and is associated with CRC progression 4. Moreover, distinct subtypes of senescent tumor cells have been identified in CRC; notably, type II senescent tumor cells are associated with local invasion and lymph node metastasis, potentially impacting patient prognosis 2. This highlights the significant role of senescence in the modulation of CRC progression.

Autophagy, a lysosomal degradation pathway, plays a critical role in the clearance of protein aggregates and damaged subcellular structures, thereby maintaining homeostasis *in vivo* 5. Autophagy and senescence are functionally related, but the role of autophagy in cellular senescence remains controversial. Some studies showed that activation of autophagy can trigger premature senescence in cancer cells 6. However, the relationship between autophagy and senescence still requires further investigation.

Chromobox homolog 8 (CBX8) is a member of the CBX protein family. Canonically, CBX8 functions as a transcriptional repressor by interacting with co-repressors such as RING1A/B and BMI1 to form the Polycomb Repressive Complex 1 (PRC1) 7, thereby facilitating chromatin compaction and gene silencing. The functions of CBX8 are complex, and previous studies have identified it as an oncogene in certain cancers. For instance, CBX8 contributes to the development and progression of bladder cancer by mediating the PRDM1/c-FOS pathway 8. CBX8 acts as a key regulator of mammary carcinoma by maintaining H3K4me3 levels on Notch-network gene promoters 9. There are a few reports on the relationship between CBX8 and tumor senescence 10. Whether CBX8 directly regulates senescence, its precise mechanisms of action, and the context-dependent signaling pathways involved constitute unresolved questions. Targeted mechanistic studies and functional validation are therefore required to elucidate these mechanisms.

In this study, we utilized a CBX8-knockout model and found that CBX8 is capable of suppressing senescence in CRC cells. CBX8 activates the mTOR pathway by transcriptionally repressing DDIT4, thereby suppressing autophagy-dependent cellular senescence. In a CRC xenograft tumor model, CBX8 inhibitors combined with senescence-targeting agents significantly enhanced the anti-tumor effect against CRC, providing additional insight into senescence-targeted anticancer therapies.

## Material and Methods

### Patient and tissue specimens

This study included patients with CRC, all of whom underwent surgery at the Department of General Surgery, Affiliated Hospital of Xuzhou Medical University. The study was reviewed and approved by the ethics committee of Xuzhou Medical University (XYFY2019-KL221-01). Written informed consent was obtained from all patients.

### Animal model

The CBX8 knockout (CBX8-KO) mice on a C57BL/6J background were generated by Cyagen Biosciences Inc. All animal-related work was approved by the Animal Center and Committee of Xuzhou Medical University. Six-week-old female BALB/C nude mice (Nanjing Jicui Pharmacom Co., Ltd.) were obtained and bred under specific pathogen-free conditions. CBX8-knockdown or control HCT116 cells (5 × 10^5^) were suspended in 100 μl of PBS and injected subcutaneously into the left side of each nude mouse (5 mice/group, randomized to the indicated groups).

### Cell lines and cell culture

The human CRC cell lines SW480 (TCHu172), DLD1 (TCHu134), and HCT116 (TCHu 99) were kindly provided by the Stem Cell Bank, Chinese Academy of Sciences. (Shanghai, China). HCT116 cells were cultured in McCoy's 5A medium (HyClone, USA) containing 10% fetal bovine serum (Gibco, USA), 100 U/ml penicillin, and 100 μg/ml streptomycin (Vicmed, China), while SW480 cells were cultured in DMEM (HyClone, Logan, UT, USA), and DLD1 cells were cultured in RPMI 1640 medium (HyClone, Logan, UT, USA). Primary cells were extracted from the colorectal cancer tissues, cells were digested by the enzymatic method, and the cells were suspended and cultured in DMEM. All cells were cultured at 37 °C in a humidified incubator with 5% CO2.

### Induction of colitis and colorectal cancer

**Figure 1:**
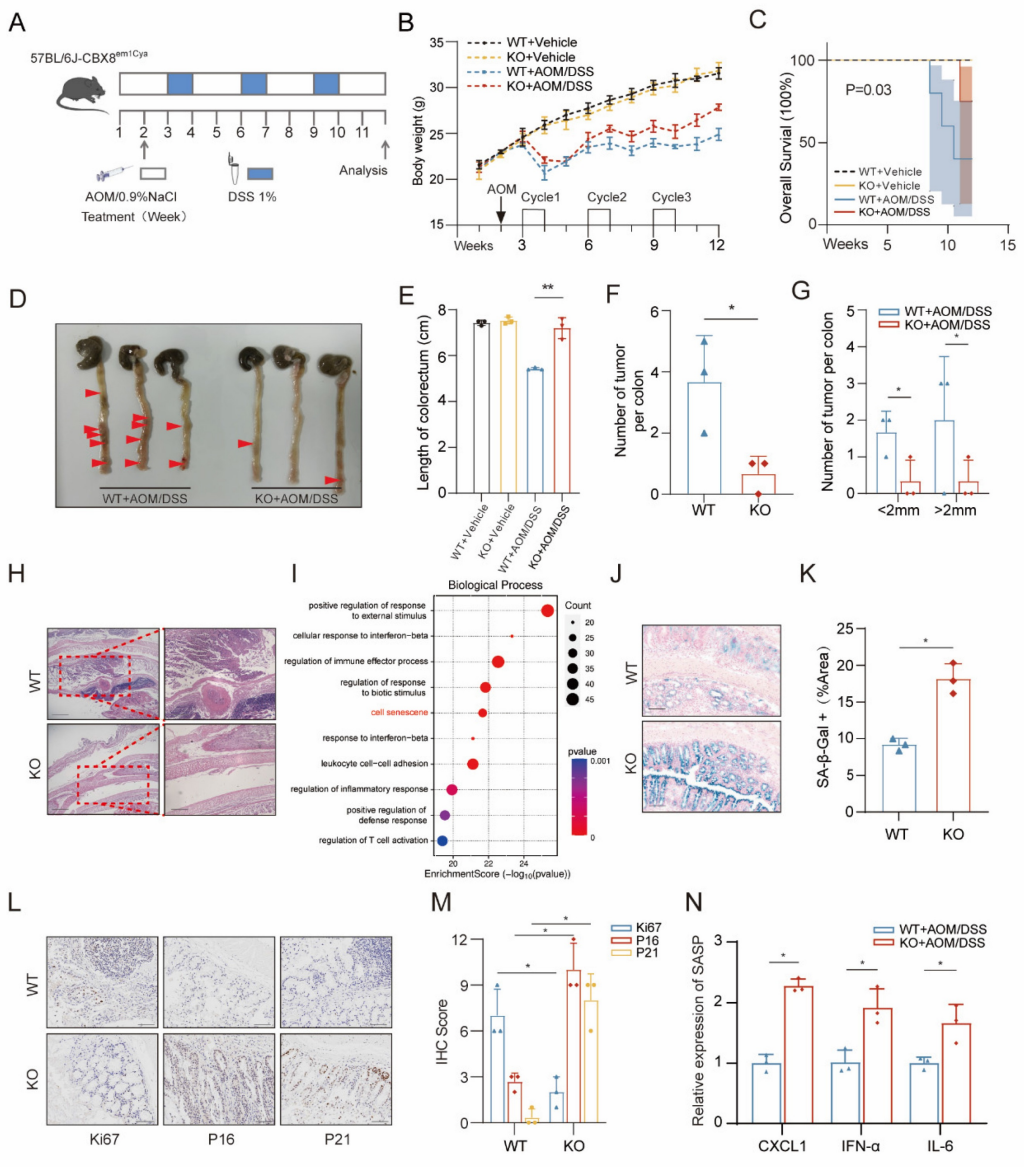
** CBX8 deficiency suppresses colorectal tumorigenesis and promotes tumor cell senescence. (A)** The mice, including CBX8 wildtype (WT) and CBX8 knockout (KO) strains, were subjected to azoxymethane (AOM) administration followed by 1% dextran sulfate sodium (DSS) treatment for three cycles as indicated. **(B)** Body weight changes are recorded in AOM/DSS-treated mice as indicated (n = 5 per genotype). **(C)** Overall survival (OS) rates are recorded in AOM/DSS-treated mice as indicated (n = 5 per genotype). **(D)** Mice colorectal tissue from representative experiments demonstrating the number and location of colon tumors (arrow). **(E)** Length of the colorectum is recorded in AOM/DSS-treated mice as indicated. **(F)** Colorectal tissue was examined, and the number of tumors was counted. **(G)** Quantifying the number and size of tumors in each mouse. **(H)** Representative histological images of AOM-DSS colorectal tumors from WT and KO mice are shown. H&E-stained sections of the mice's distal colon tissue are shown. **(I)** Bubble chart showing the KEGG analysis of differentially expressed genes in response to CBX8 knockout.** (J-K)** Representative images of SA-β-Gal staining in the intestine of mice in the indicated groups, and the number of SA-β-Gal-positive cells in each group was quantified. **(L-M)** Representative images of Ki67, P16 and P21 staining in AOM-DSS colorectal tumors from WT and KO mice are shown, and IHC scores were quantified (n = 3 per genotype). **(N)** Quantification of the abundance of the indicated mRNAs in mouse tumor tissues. The data represent the findings from three independent experiments and are shown as the means±SDs (*, *p*<0.05; **, *p*<0.01, ***, *p*<0.001). Scale bars: 100 μm (right, **h; j**), 150 μm (left, **h**), 200 μm (**j**).

For the azoxymethane (AOM)/dextran sulfate sodium (DSS) model, we followed the published protocol with modifications (Figure 1A). Briefly, CBX8 knockout (KO) and CBX8 wild-type (WT) male mice (age, 7-8 weeks) were injected intraperitoneally with AOM (10 mg/kg body weight). 7 days after AOM administration, mice were given 1% DSS water for 7 days, followed by regular water for 14 days. The DSS treatments were repeated 3 times. Mice were killed after 12 weeks for further analysis. Mice were weighed/observed daily during DSS treatment. AOM was purchased from Sigma. DSS was purchased from Thermo Fisher Scientific (Waltham, MA).

### Short interfering RNA (siRNA), short hairpin RNA (shRNA) and plasmid construction, plasmid transfection and RNA interference

CRC cells were transfected with siRNA (GenePharma, China) using siLentFect lipid reagent (Bio-Rad, USA) and were infected with plasmid (Genechem, China) using Lipofectamine 3000 transfection reagent (ThermoFisher, USA) according to the manufacturer's protocol. pSuper.retro.puro retroviral constructs containing short hairpin RNA (shRNA) against human CBX8 were prepared by GenePharma, Shanghai, China. Infected cells were selected by adding 2 μg/ml puromycin to the culture medium for 48 h and then maintained in complete medium with 0.5 μg/ml puromycin. The transfection effects were verified by real-time quantitative reverse transcription PCR (qRT‒PCR) and Western blotting (WB). All sequences are provided in the Supplementary Materials and Methods.

### Immunoprecipitation

Immunoprecipitation was performed using the Pierce™ Classic Magnetic IP/Co-IP kit (Thermo) according to the manufacturer's protocol. Cells were lysed in lysis buffer (50 mM HEPES, pH 7.4, 150 mM NaCl, 1% Triton X-100, 10% glycerol, 2 mM MgCl2, 2 mM EGTA) with protease inhibitor and phosphatase inhibitor mixture (Thermo) on ice. Cell lysates were centrifuged to remove insoluble material. Antibodies were incubated with magnetic beads overnight at 4°C. Beads were washed repeatedly and bound proteins were analyzed by protein blotting.

### Immunohistochemistry

Immunohistochemical (IHC) staining was performed on paraffin-embedded CRC tissue specimens and mouse tissue specimens. Briefly, after baking and deparaffinization, tissue sections were stained with antibodies followed by anti-rabbit horseradish peroxidase-coupled secondary antibodies. Relevant gene expression was assessed by a specialized pathologist. IHC staining scores were calculated based on the percentage of positive tumor cells and staining intensity.

### SA-β-gal staining

Cells were fixed using a PBS solution of 2% formaldehyde and 0.2% glutaraldehyde and washed twice with PBS. Cells were stained in a non-CO2 incubator at 37 °C using the SA-β-gal staining kit as directed. For mouse tumor tissue, fix frozen sections and stain at pH 5.5 for 5- 8 hours.

### Chromatin immunoprecipitation (ChIP) assay

Cells were cross-linked with 1% formaldehyde and then terminated with 125 mmol/L glycine. The cells were then lysed and the DNA was digested to approximately 200 bp by enzymatic dissociation, followed by immunoprecipitation with ChIP-grade antibody or IgG. Purified DNA was isolated for qRT-PCR using a DNA purification kit.

### Transmission electron microscopy

Treated CRC cells were incubated with pre-cooled 2% glutaraldehyde solution for 2 hours at 4°C to fix the cell precipitate. Cells were stained with 2% uranyl acetate solution for 2 hours and then dehydrated in 50%, 70%, 90% and 100% acetone. Cells were embedded in a Spurr embedding kit and ultrathin sections were prepared for observation under an electron microscope (HITACHI).

### Gene set enrichment analysis (GSEA)

GSEA was performed on the normalized data using the GSEA v2.0 tool (http://www.broad.mit.edu/gsea/). We compared the gene expression between cells with high and low CBX8 expression. Gene sets were used for GSEA. The p-values of the differences between the two gene sets were analyzed with the Kolmogorov-Smirnov test.

### Statistical methods

Statistical analyses were performed using SPSS 20.0 software (IBM, Armonk, NY, USA) and the mean±standard deviation was used to express the normally distributed measures. Student's t-test was used to evaluate the significance of differences between groups. A P value of < 0.05 was considered statistically significant.

## Results

### CBX8 deficiency suppresses colorectal tumorigenesis and promotes tumor cell senescence

Previous studies have identified CBX8 as an oncogene in cancer biotherapy 11. Given the well-established link between chronic colitis and CRC, we investigated the role of CBX8 in CRC susceptibility using a CBX8 knockout (KO) mouse model in the AOM/DSS-induced CRC model 12. To elucidate the functional significance of CBX8 in CRC, we generated CBX8 knockout (CBX8 KO; CBX8(-/-)) mice by deleting the CBX8 genomic sequence. Genomic deletion in CBX8 KO mice was confirmed by PCR analysis (Figure S1A). To induce colorectal tumorigenesis, mice were administered 10 mg/kg AOM via intraperitoneal injection, followed by three cycles of 1% DSS treatment (Figure 1A). At week 12 post-induction, the mice were euthanized, and their intestines were collected for analysis. During DSS treatment, CBX8 KO mice exhibited reduced body weight loss (Figure 1B), improved survival rates with 95% confidence intervals (Figure 1C; KO: 5.3%-85.3% vs. WT: 28.4%-99.5%), and longer colorectal length compared to wild-type (WT) CBX8 mice (CBX8 WT; CBX8(+/+)) (Figure 1D-E), suggesting that CBX8 deficiency confers resistance to DSS-induced colitis. Notably, CBX8 KO mice developed significantly fewer colorectal and distal colon tumors compared to CBX8 WT mice (Figure 1F-G). Histopathological analysis further confirmed a marked reduction in tumor burden across different size categories, as well as an earlier disease stage in CBX8 KO mice (Figure 1H).

To further explore the molecular mechanisms underlying this phenotype, we performed deep sequencing of colorectal tumors from 3-month-old CBX8 WT and KO mice. KEGG enrichment analysis of the transcriptomic data indicated that CBX8 is involved in tumor cell senescence (Figure 1I). To investigate the relationship between CBX8 and cellular senescence in CRC development, we performed senescence-associated β-galactosidase (SA-β-gal) staining, a lysosomal enzyme commonly used as a marker of senescence 13, on intestinal tissues from AOM/DSS-treated mice. CBX8 KO mice exhibited a significantly higher proportion of SA-β-gal-positive cells compared with CBX8 WT controls (Figure 1J-K). We performed immunohistochemical (IHC) analysis, which revealed that CBX8 KO tumors exhibited reduced proliferation, as indicated by lower Ki67 staining, and increased expression of senescence-associated markers, including P21 and P16 14, compared to CBX8 WT (Figure 1L-M). Cellular senescence is characterized by a distinct secretory profile known as the senescence-associated secretory phenotype (SASP) 15. Quantitative analysis revealed that key SASP components, including CXCL1, IFN-α, and IL-6, were significantly upregulated in CBX8-deficient tumors relative to WT tumors (Figure 1N). Consistently, heatmap analysis showed significantly elevated expression of key senescence-associated genes, including CDKN1A, CDKN2A, LMNB1, and PCK1, in CBX8-deficient tumors (Figure S1B). These findings suggest that CBX8 loss may contribute to CRC suppression by promoting tumor cell senescence.

### Cellular senescence induction in CRC via CBX8 downregulation

**Figure 2:**
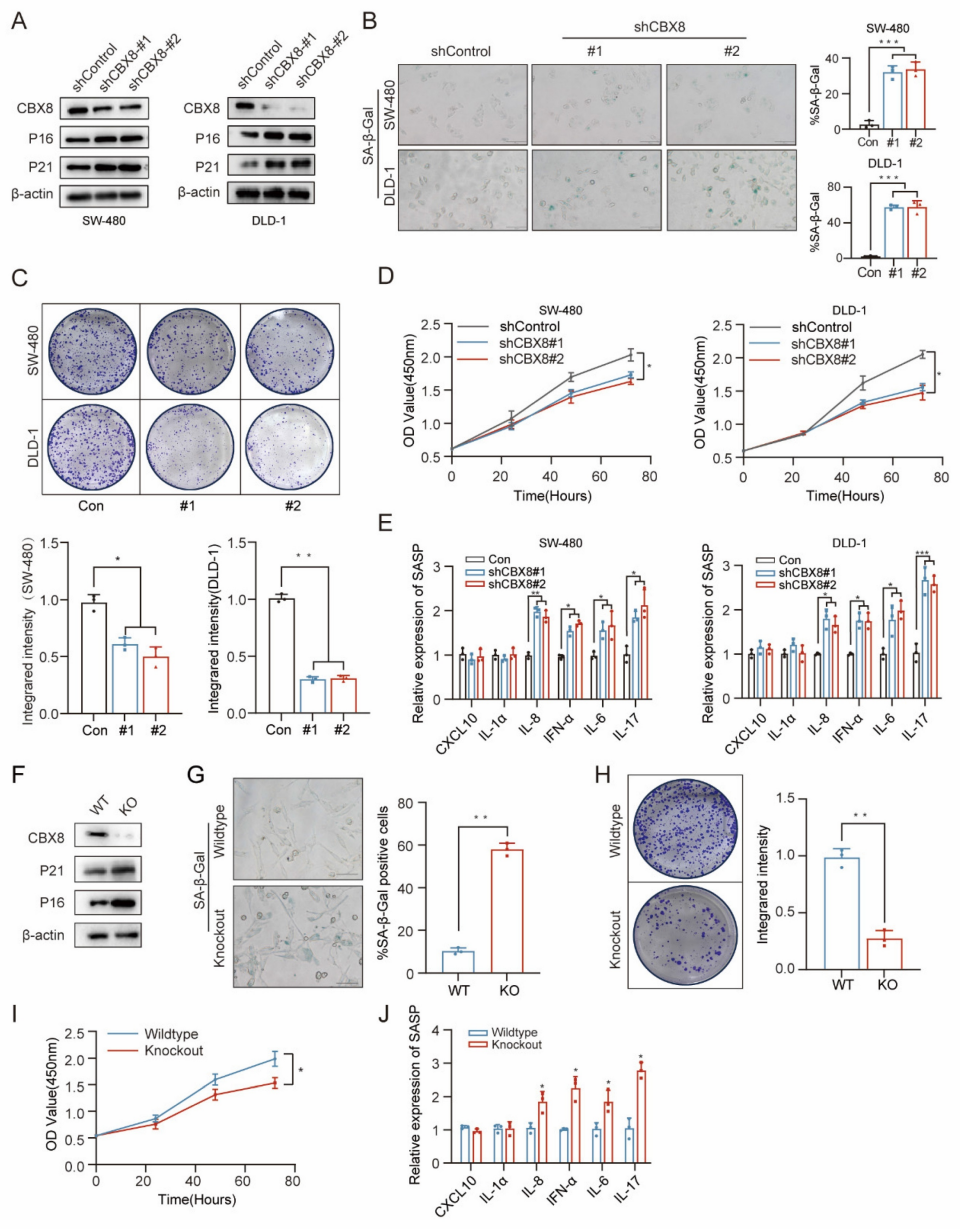
** Cellular senescence induction in CRC via CBX8 downregulation. (A)** Expression of the indicated proteins was analyzed by immunoblotting in SW480 (left) and DLD-1 cells (right). The experiment was repeated three times independently with similar results. **(B)** CRC cells expressing shControl or shCBX8 were stained for SA-β-Gal activity as indicated, and the percentage of SA-β-Gal-positive cells in the normal and knockdown groups was quantified in the right panel.** (C)** Colony formation assays were performed on SW480 and DLD-1 cells to determine senescence-associated growth arrest and the integrated intensity for each group was quantified in the bottom panel.** (D)** Cell viability assays were performed on SW480 and DLD-1 cells to determine senescence-associated growth arrest.** (E)** Quantification of the abundance of the indicated mRNAs in SW480 and DLD-1 cells transfected with the indicated shRNAs. The relative abundance of the indicated mRNAs is expressed as a change in expression in cells under the empty vector control. **(F)** Expression of the indicated proteins was determined by immunoblotting in intestinal primary cells expressing CBX8 wildtype or knockout. **(G)** The representative images of primary culture cells in AOM-DSS colorectal tumors from wildtype and knockout mice were harvested and stained for SA-β-Gal activity. **(H)** Colony formation assays were performed on primary culture cells to determine senescence-associated growth arrest and the integrated intensity for each group was quantified in the right panel. **(I)** Cell viability assays were performed on primary culture cells to determine senescence-associated growth arrest.** (J)** Quantification of the abundance of the indicated mRNAs in primary culture cells. The data represent the findings from three independent experiments and are shown as the means±SDs (*, *p*<0.05; **, *p*<0.01, ***, *p*<0.001). Scale bars: 100 μm (**b, g**).

To determine whether CBX8 downregulation is sufficient to induce senescence, we established stable CBX8-knockdown human colon cancer cell lines (SW-480 and DLD-1) using two independent short hairpin RNAs (shRNAs). Western blot analysis confirmed that CBX8 knockdown led to increased expression of senescence markers, including P16 and P21 (Figure 2A). CBX8 knockdown led to increased SA-β-Gal activity and inhibited cell growth (Figure 2B-D). To assess SASP following CBX8 depletion, qPCR analysis was performed, revealing elevated expression of IL-8, IFN-α, IL-6, and IL-17 in CBX8-deficient cells (Figure 2E). Similarly, intestinal tumor cells isolated from CBX8-knockout mice exhibited a significant increase in P16 and P21 expression, SA-β-Gal activity, and IL-8, IFN-α, IL-6, and IL-17 expression, along with reduced proliferative capacity (Figure 2F-J).

To further validate the role of CBX8 in regulating tumor cell senescence, CRC cells were treated with SW2_110A, a targeted CBX8 inhibitor 16. IC50 of SW-480 cell was 52.53 μM and IC50 of DLD-1 cell was 32.88μM (Figure S1C). CBX8 expression was significantly suppressed at 24 hours (Figure S1D), accompanied by the upregulation of senescence markers similar to those observed in shCBX8-treated cells (Figure S1E-F). Additionally, senescence-associated cell proliferation was inhibited significantly following SW2_110A treatment (Figure S1G-H), along with alterations in the SASP (Figure S1I). In conclusion, these findings demonstrate that CBX8 downregulation promotes senescence *in vitro*.

### CBX8 suppresses autophagy-dependent senescence in CRC cells

**Figure 3:**
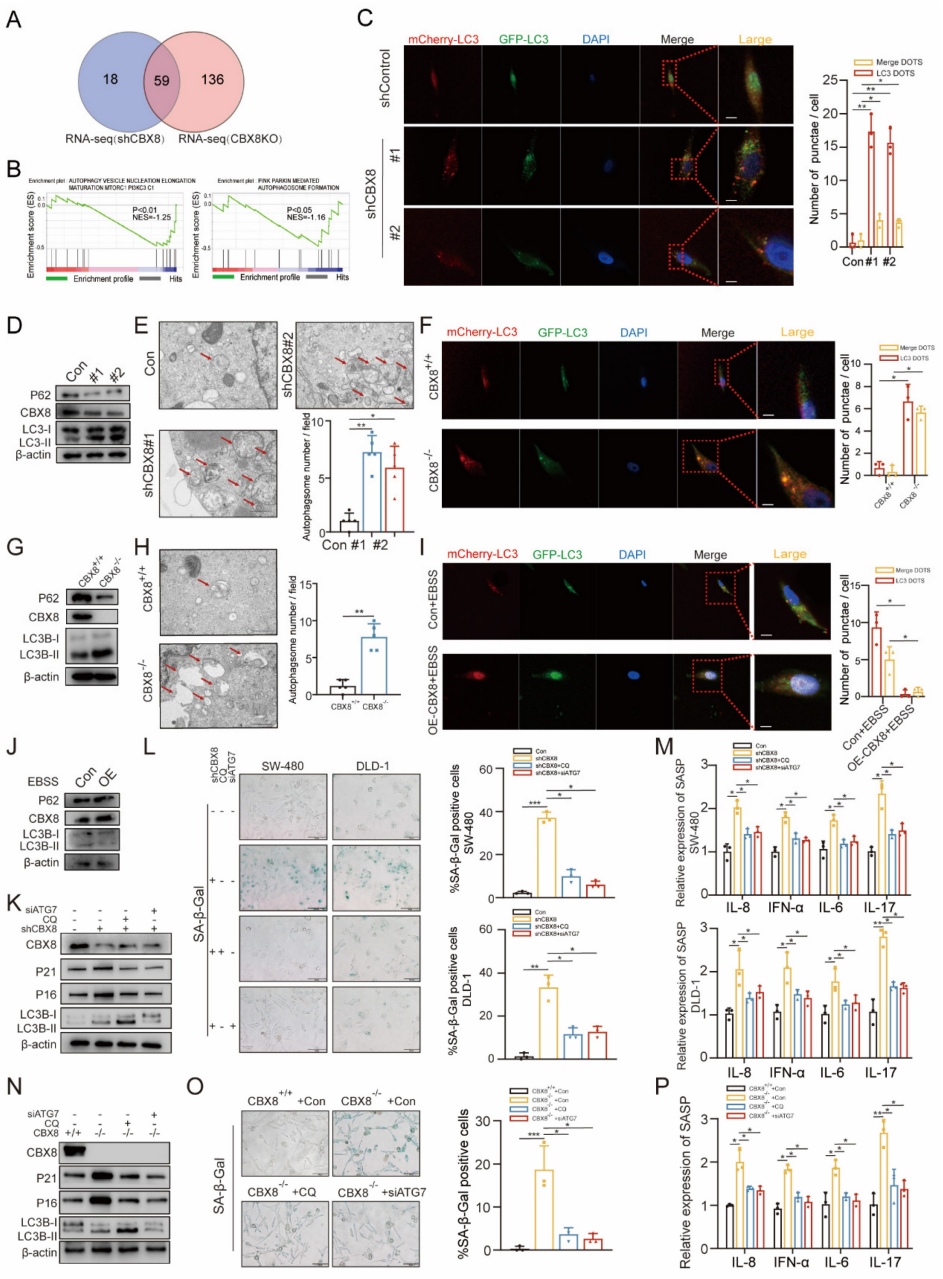
** CBX8 suppresses autophagy-dependent senescence in CRC cells. (A)** Venn diagram showing the number of GSEA analyses of transcriptome differential genes in shCBX8 and CBX8-KO. **(B)** GSEA showing that the genes that were differentially expressed after CBX8 knockdown (left) and knockout (right) were enriched in the autophagy related gene set.** (C)** The effect of mCherry-GFP-LC3 point distribution in DLD-1 cells 48 hours after mCherry-GFP-LC3 plasmid transfection is shown by confocal laser scanning microscopy. The bar graph above showed the number of autophagosomes and the number of LC3 spots.** (D)** Immunoblotting (IB) of the indicated protein in shCBX8 cells. **(E)** Autophagosome detection by transmission electron microscopy (TEM) and quantification in the DLD-1 cells. The arrow indicates the autophagosome. **(F)** The effect of mCherry-GFP-LC3 point distribution in CBX8 knockout cells 48 hours after mCherry-GFP-LC3 plasmid transfection is shown by confocal laser scanning microscopy. The bar graph above showed the number of autophagosomes and the number of LC3 spots.** (G)** IB of the indicated protein in the CBX8 knockout cells. **(H)** Autophagosome detection by TEM and the quantification in the CBX8 knockout cells. The arrow indicates the autophagosome. **(I)** The effect of mCherry-GFP-LC3 point distribution in DLD-1 cells 48 hours after mCherry-GFP-LC3 plasmid transfection is shown by confocal laser scanning microscopy. The bar graph above showed the number of autophagosomes and the number of LC3 spots.** (J)** IB of the indicated protein in the CBX8 OE cells. **(K)** IB analysis of the indicated protein in DLD-1 cells with or without CQ treatment (10 µM) for 24h or siATG7 transfection.** (L)** CRC cells expressing shControl or shCBX8 were stained for SA-β-Gal activity after treatment with CQ (10 µM) for 24h or siATG7 transfection. Quantification of the percentage of SA-β-Gal positive cells. **(M)** Results of quantification of the abundance of the indicated mRNAs in CRC cells transfected with or without CQ application or siATG7 transfection. The relative abundance of the indicated mRNAs is expressed as the change in expression in cells under empty vector control.** (N)** IB analysis of the indicated protein in WT or KO with or without CQ (10 µM) treatment. **(O)** The CBX8 knockout cells were stained for SA-β-Gal activity after treatment with CQ (10 µM) for 24h or siATG 7 transfection and quantification of the percentage of SA-β-Gal positive cells. **(P)** Results of quantification of the abundance of the indicated mRNAs in the CBX8 knockout cells transfected with or without CQ application or siATG7 transfection. The experiment was repeated three times independently with similar results. The data represent the findings from three independent experiments and are shown as the means±SDs (*, *p*<0.05; **, *p*<0.01, ***, *p*<0.001). Scale bars: 200 nm (**e, h**),10 μm (**c, f, i**),100 μm (**l, o**).

To elucidate the molecular mechanisms underlying CBX8-mediated regulation of senescence, we performed RNA-seq analysis on CBX8-depleted CRC cells. Integrating these results with RNA-seq data from CBX8 knockout mice, we identified a shared set of differentially expressed genes that were significantly enriched in autophagy related pathways (Figure 3A-B). As shown in the heatmap, key autophagy-regulating genes, including ATG16L1, HDAC6, SQSTM1, and ATG4D, were significantly changed following CBX8 downregulation in both RNA-seq datasets (Figure S2A), suggesting that autophagy activation occurs in the absence of CBX8.

To test this hypothesis, the autophagic flux rate was measured using an mCherry-GFP-LC3 reporter construct. CBX8 knockdown resulted in a significant increase in the number of autophagosomes (yellow puncta) and autolysosomes (red puncta) (Figure 3C, Figure S2B). Western blot analysis further confirmed an elevated LC3-II/LC3-I ratio, a reduction in p62 levels and an upregulation in ATG5 and Beclin1 levels following CBX8 depletion (Figure 3D, Figure S2C). Transmission electron microscopy revealed an increased number of autophagosomes and autolysosomes in CBX8-knockdown CRC cells (Figure 3E). Consistent findings were observed in CBX8-KO mouse cells (Figure 3F-H, Figure S2D). Conversely, CBX8 overexpression inhibited autophagy, further supporting its role as a negative regulator of autophagy in CRC (Figure 3I-J, Figure S2F).

Senescence is a dynamic process involving multiple effector mechanisms. It has been reported that autophagy is activated during senescence and plays a crucial role in facilitating the acquisition of the senescence phenotype 17. To clarify the relationship between senescence and autophagy in CRC cells, chloroquine (CQ, 10 µM), a lysosomal degradation inhibitor, and ATG7 siRNA were used to disrupt autophagic flux in CBX8-knockdown CRC cells. The increased expression of senescence markers and SA-β-Gal activity observed in CBX8-knockdown cells was reversed by CQ and siATG7 treatment (Figure 3K-L, Figure S2G-H). Furthermore, the upregulation of SASP factors induced by CBX8 knockdown was attenuated following CQ and siATG7 treatment (Figure 3M). A similar autophagy-mediated reversal of the senescence phenotype was observed in CBX8-KO mouse cells (Figure 3N-P), reinforcing the role of autophagy in CBX8-mediated senescence regulation.

### CBX8 regulates autophagy-dependent senescence in colorectal cancer through DDIT4-mediated modulation of mTOR signaling

**Figure 4:**
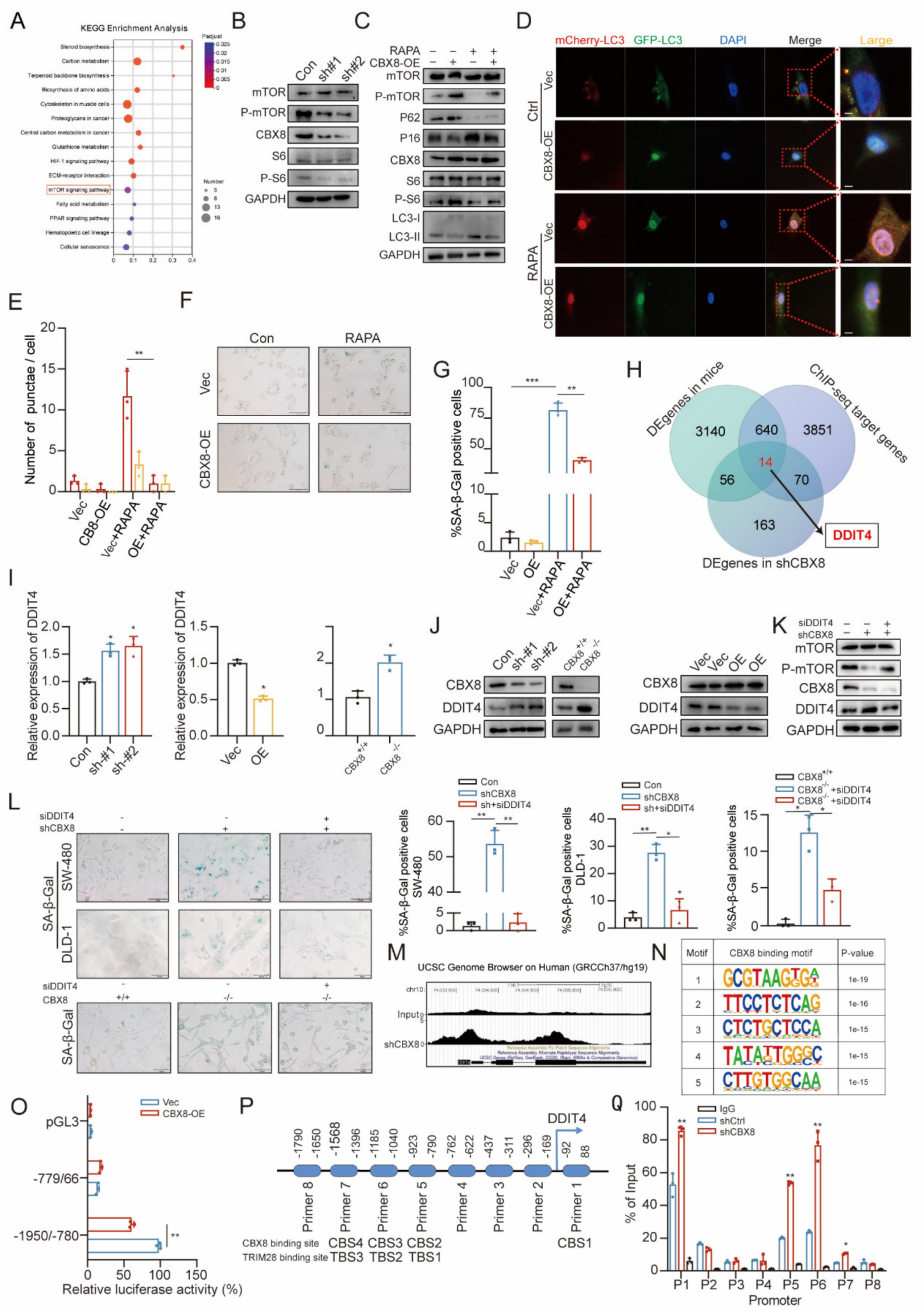
** CBX8 regulates autophagy-dependent senescence in colorectal cancer through DDIT4-mediated modulation of mTOR signaling. (A)** Bubble chart showing the KEGG analysis of differentially expressed genes in response to CBX8 knockdown. **(B)** Immunoblotting of the indicated protein level.** (C)** IB showing the effects of CBX8 overexpression on the indicated protein levels in SW480 cells treated with rapamycin (RAPA,10 nM). **(D-E)** The effect of mCherry-GFP-LC3 point distribution in SW480 cells 48 hours after mCherry-GFP-LC3 plasmid transfection is shown by confocal laser scanning microscopy.** (F-G)** SW480 cells overexpressing CBX8 were stained for SA-β-Gal activity, treated with or without RAPA as indicated, and the percentage of SA-β-Gal-positive cells in each group is quantified.** (H)** Venn diagram showing overlapping genes in CBX8 knockout mice colorectal tumor transcriptome, shCBX8 knockdown human colorectal cancer cell line transcriptome and CBX8 knockdown ChIP data.** (I)** The expression levels of DDIT4 were detected via qRT-PCR analysis. GAPDH was the internal control.** (J)** IB analysis of the indicated protein in shControl or shCBX8, CBX8 wildtype or CBX8 knockout and CBX8 empty vector or CBX8 overexpression.** (K)** IB showing the effects of CBX8 knockdown on the indicated protein levels in DLD-1 cells treated with siDDIT4.** (L)** CRC cells expressing shControl or shCBX8 and CBX8 wildtype or CBX8 knockout with or without siDDIT4 were stained for SA-β-Gal activity and quantified for percentage of positive cells in the right panel. **(M)** Gene annotation information in the UCSC database indicates the CBX8 peak line of the DDIT4 promoter.** (N)** The top five predicted CBX8-binding elements were obtained by de novo motif analysis using HOMER software. **(O)** Luciferase reporter genes driven by the -1950/-780 or - 770/66 fragments of the DDIT4 promoter region were cotransfected into SW480 cells, and luciferase activity was measured after 48 hours. The relative luciferase activity value in cells cotransfected with pRL-TK (-1950/-780) and shCtrl was set to 100%. **(P)** A schematic of the eight DDIT4 promoter regions (1-8) analyzed for CBX8 binding affinity (above). Schematic representation of predicted TRIM28 with DDIT4 binding sites (below).** (Q)** ChIP-qPCR analysis was used to determine the binding affinity of CBX8 to 8 DDIT4 promoter regions in DLD-1 cells, showing that CBX8 bound to the CBS1-CBS4 regions in the DDIT4 promoter. ChIP-qPCR with IgG was performed as the control. The experiment was repeated three times independently with similar results. The data represent the findings from three independent experiments and are shown as the means±SDs (*, *p*<0.05; **, *p*<0.01, ***, *p*<0.001). Scale bars: 10 μm (**d**),100 μm (**f, l**).

To investigate the specific mechanism by which CBX8 regulates autophagy-dependent senescence in colorectal cancer, we performed KEGG analysis on RNA-seq data from CBX8-knockdown cells. The analysis revealed that the mTOR signaling pathway was significantly affected (Figure 4A). In addition, enrichment of the mTOR signaling pathway was similarly observed in the mouse RNA-seq data (Figure S3A). Studies have reported that the mTOR pathway plays a critical role in regulating fundamental cellular processes, including autophagy and senescence 18, 19. We examined whether it is involved in CBX8-induced autophagy-dependent senescence in CRC cells. Western blot analysis showed that CBX8 knockdown in DLD-1 cells led to reduced phosphorylation levels of mTOR and its downstream target S6K (Figure 4B, Figure S3B). We treated CBX8-overexpressing SW-480 cells with the mTOR inhibitor rapamycin (10 nM) and found that mTOR inhibition not only reversed the upregulation of p-mTOR and p-S6 but also restored autophagy activity (Figure 4C-E) and the suppressed senescence phenotype (Figure 4F-G). These results indicate that CBX8 modulates autophagy and senescence in CRC cells by regulating the mTOR signaling pathway. To further elucidate the molecular mechanism by which CBX8 regulates the mTOR pathway in colorectal cancer cells, we performed ChIP-seq analysis to identify genome-wide CBX8 binding sites. A total of 22,917 peaks corresponding to 4,575 RefSeq genes were identified, with CBX8 predominantly enriched near transcription start sites (TSSs) (Figure S3C). By integrating ChIP-seq data with differentially expressed genes from CBX8 knockdown and CBX8 KO models, we identified 14 overlapping genes (Figure 4H, Figure S3D). Notably, DDIT4, a well-known negative regulator of mTOR, was among these genes, suggesting a potential regulatory link between CBX8 and mTOR signaling via DDIT4.

qPCR and Western blot analyses confirmed that CBX8 exerts a negative regulatory effect on DDIT4 expression (Figure 4I-J, Figure S3E-F). We then transfected CBX8-silenced CRC cells with DDIT4 siRNA. Depletion of DDIT4 reversed the reduction in p-mTOR levels and the increase in SA-β-Gal activity induced by CBX8 knockdown in both CRC cell lines and CBX8-KO mouse cells (Figure 4K-L, Figure S3G-J). Further validation of the direct link between CBX8 and DDIT4 was performed, ChIP-seq results showed that CBX8 was enriched at the DDIT4 promoter (Figure 4M). HOMER motif analysis identified the top five predicted binding motifs, with the first motif enriched in the DDIT4 promoter, suggesting its potential role in transcriptional regulation (Figure 4N). To assess whether DDIT4 is regulated by CBX8 through promoter binding, we constructed dual-luciferase reporter plasmids containing DDIT4 promoter regions. Luciferase assays revealed that CBX8 knockdown significantly enhanced luciferase activity driven by the -1950/-780 promoter region, while CBX8 overexpression reduced activity (Figure 4O, Figure S3K-L). Additionally, we designed eight primer pairs targeting different DDIT4 promoter regions (regions 1-8, Figure 4P). ChIP-qPCR analysis demonstrated that silencing CBX8 reduced its binding to regions 1, 5, 6, and 7 (CBS1-4) of the DDIT4 promoter, while CBX8 overexpression enhanced binding to these regions (Figure 4Q, Figure S3M-N). Confirming that CBX8 directly regulates the DDIT4 transcription by binding to specific promoter regions.

### CBX8 maintains the H3K27me3 status at the DDIT4 promoter by binding to TRIM28

**Figure 5:**
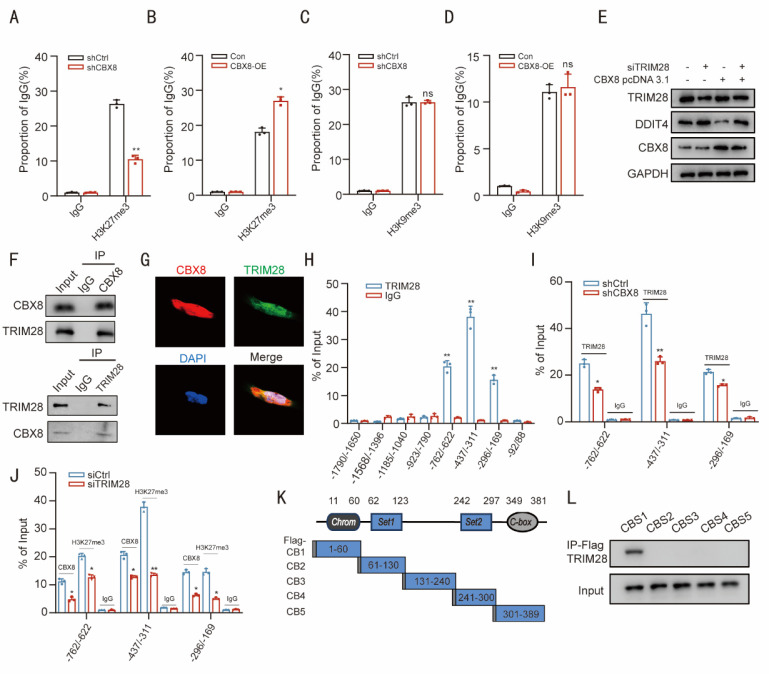
** CBX8 maintains the H3K27me3 status at the DDIT4 promoter by binding to TRIM28. (A)** Enrichment of H3K27me3 at DDIT4 promoter in DLD-1 cells expressing empty vector (shCtrl) or shCBX8. ChIP-qPCR with IgG was performed as the control. **(B)** Enrichment of H3K27me3 in the DDIT4 promoter region in SW480 cells overexpressing CBX8 or empty vector. ChIP-qPCR with IgG was performed as the control. **(C)** Enrichment of H3K9me3 at DDIT4 promoter in DLD-1 cells expressing empty vector (shCtrl) or shCBX8. ChIP-qPCR with IgG was performed as the control. **(D)** Enrichment of H3K9me3 in the DDIT4 promoter region in SW480 cells overexpressing CBX8 or empty vector. ChIP-qPCR with IgG was performed as the control. **(E)** Western blot analysis of DDIT4 expression following TRIM28 knockdown in SW480 cells overexpressing CBX8. **(F)** CoIP showed the interaction between CBX8 and TRIM28 proteins in HEK293 cells.** (G)** The location of CBX8 and TRIM28 in DLD-1 cells. **(H)** ChIP-qPCR analysis performed to determine the binding affinity of TRIM28 to the eight DDIT4 promoter regions in DLD-1 cells showed that TRIM28 bound to the TBS1-TBS3 regions in the DDIT4 promoter. ChIP- qPCR with IgG was performed as the control. **(I)** ChIP-qPCR analysis was used to determine the binding affinity of TRIM28 to the TBS1-TBS3 regions after CBX8 knockdown. **(J)** ChIP-qPCR analysis showed the enrichment of CBX8 and H3K27me3 in the TBS1-TBS3 regions after TRIM28 knockdown in DLD-1 cells. **(K)** Schematic of the five Flag-CBX8 recombinant proteins (CB1-CB5). **(L)** Plasmids encoding a Flag-tagged, CBX8 truncation mutant were transfected into DLD-1 cells, and anti-Flag antibody was used to immunoprecipitate the bound proteins. The TRIM28 level in the immunoprecipitates was determined by Western blot. The experiment was repeated three times independently with similar results. The data represent the findings from three independent experiments and are shown as the means±SDs (*, *p*<0.05; **, *p*<0.01, ***, *p*<0.001). Scale bars: 10 μm (**g**).

Previous studies have demonstrated that CBX8 plays a crucial role in transcriptional regulation through its affinity for methylated histone peptides, specifically H3K27me3 and H3K9me3 20^.^ ChIP assays revealed that CBX8 depletion decreased H3K27me3 levels at the DDIT4 promoter, while CBX8 overexpression increased H3K27me3 (Figure 5A-B, Supplementary Figure 4A). However, CBX8 silencing did not affect H3K9me3 (Figure 5C-D, Figure S4B). These results suggest that CBX8 regulates DDIT4 expression through H3K27me3 modifications.

Recent studies have reported that CBX proteins interact with cofactors to modulate epigenetic landscapes, leading to the activation or repression of gene expression 9. We then performed immunoprecipitation (IP) assays followed by mass spectrometry (MS) analysis to identify potential CBX8-interacting proteins. A total of 40 candidate proteins were co-precipitated (Table S1), including TRIM28, a nuclear corepressor involved in gene expression (Transcription) 21. We investigated whether TRIM28 participates in CBX8-induced DDIT4 activation. Western blot analysis revealed that TRIM28 knockdown suppressed the DDIT4 inhibition induced by CBX8 overexpression (Figure 5E). We performed additional TRIM28 KO experiments in CRC cells using CRISPR-Cas9 (Figure S4C). WB analysis showed that knockout of TRIM28 alone resulted in significant upregulation of DDIT4, decreased levels of p-mTOR, increased LC3-II, and increased expression of senescence markers p21. Re-expression of CBX8 in TRIM28-KO cells had minimal effect on DDIT4 expression, mTOR activation, autophagy, or senescence markers, confirming that TRIM28 is essential for CBX8-mediated regulation of the DDIT4/mTOR pathway (Figure S4D). Additionally, Co-immunoprecipitation (Co-IP) assays demonstrated a reciprocal interaction between CBX8 and TRIM28 in DLD-1 cells. This interaction was further confirmed in HEK293 cells, where co-expression of these labeled proteins resulted in CoIP detection (Figure 5F, Figure S4E). The co-localization of CBX8 and TRIM28 was further examined through immunofluorescence analysis (Figure 5G).

We re-examined regions 1-8 of the DDIT4 promoter through ChIP. The ChIP assay revealed that TRIM28 was significantly enriched at regions 5, 6, and 7. CBX8 knockdown reduced TRIM28 occupancy at these regions, while TRIM28 depletion diminished the enrichment of both CBX8 and H3K27me3 at regions 5, 6, and 7 (Figure 5H-J). These results suggest that CBX8 facilitates the accumulation of TRIM28 at regions 5, 6, and 7 to sustain the H3K27me3 modification status. To identify the CBX8 binding domain responsible for interacting with DDIT4, we cloned five Flag-tagged CBX8 constructs representing different regions (Figure 5K) and transfected them into DLD-1 cells. Cell extracts were then subjected to immunoprecipitation (IP) using an anti-Flag antibody. The Flag-CBX8-1 construct was the only one to interact with TRIM28 in cancer cells (Figure 5L), suggesting that the CBX8-1 domain, spanning amino acids 1 to 60, is crucial for its interaction with TRIM28.

### CBX8 depletion promotes cellular autophagy and senescence *in vivo*

**Figure 6:**
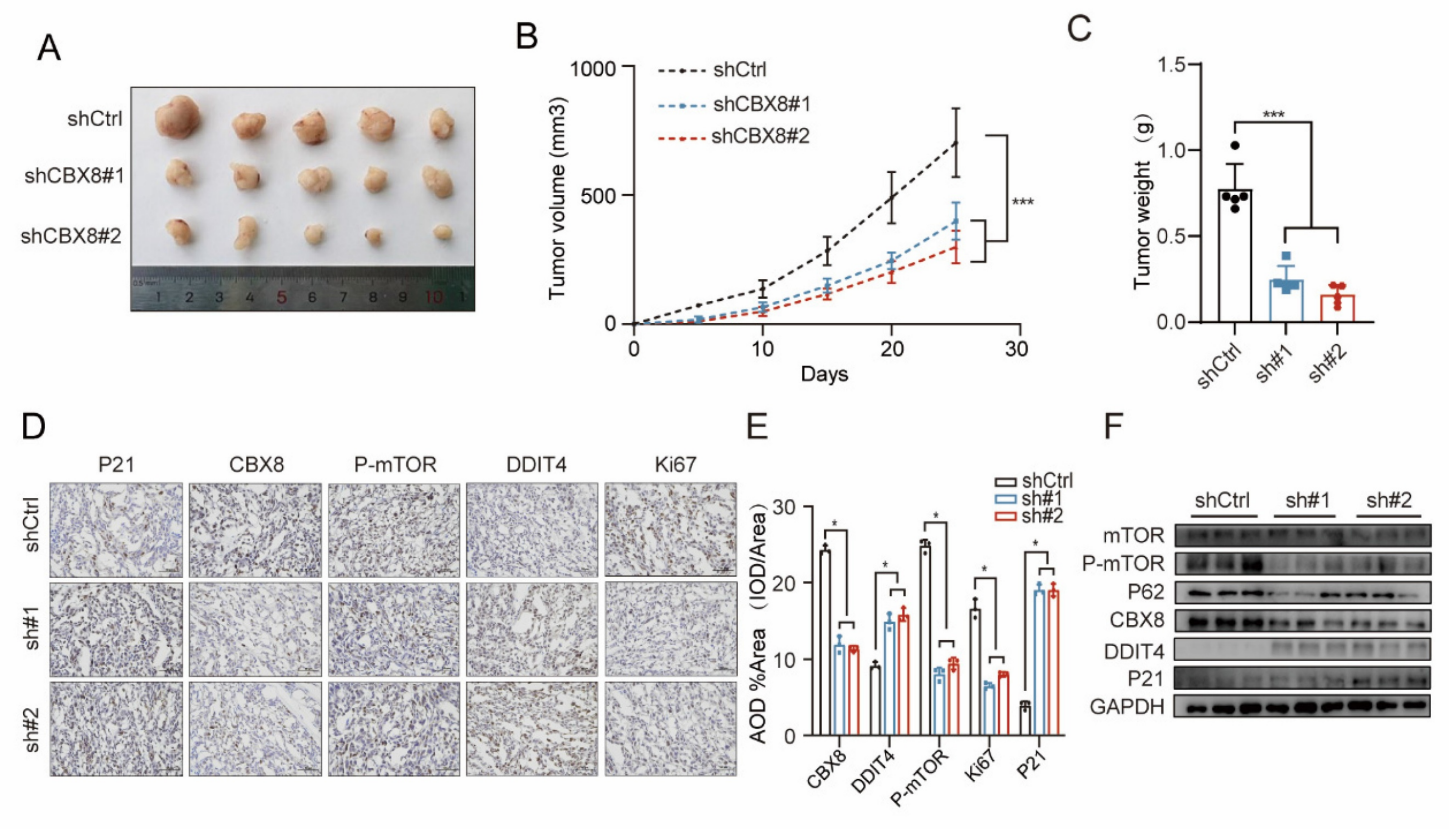
** CBX8 depletion promotes cellular autophagy and senescence *in vivo*. (A)** Representative images of xenograft tumors in nude mice after different treatments. shCtrl or shCBX8 were injected intratumorally. **(B)** The tumor growth curves of each group of mice are summarized. **(C)** Tumor weights were examined in CDX as indicated. (n = 5 per group). **(D-E)** IHC staining for P21, Ki67, P-mTOR, DDIT4 and CBX8 in the indicated groups was shown (×400 magnification) and AOD values were quantified. **(F)** Western blot analysis of the indicated protein in control or shCBX8. The experiment was repeated three times independently with similar results. The data represent the findings from three independent experiments and are shown as the means±SDs (*, *p*<0.05; **, *p*<0.01, ***, *p*<0.001). Scale bars: 50 μm (right, **d**).

To further investigate the role of CBX8 in autophagy and senescence in a mouse xenograft model, we injected nude mice with either vector control CRC cells or CRC cells with stable CBX8 knockdown. Tumor growth was significantly inhibited in the CBX8 knockdown group compared to the control group, as evidenced by a marked reduction in tumor size and weight at the end of the evaluation period (Figure 6A-C).

IHC analysis revealed strong staining for DDIT4 and P16, along with weak Ki-67, p62 and p-mTOR staining, in xenograft tumors with CBX8 knockdown compared to the control group (Figure 6D-E). Furthermore, Western blot analysis confirmed that stable CBX8 knockdown led to a marked downregulation of p-mTOR, p62 and upregulation of DDIT4, and P16 (Figure 6F). In summary, down-regulation of CBX8 *in vivo*, which promotes cellular autophagy and senescence, exerts anti-tumor effects.

### Enhancement of antitumor effects in CRC by CBX8 inhibitors in combination with senescence scavengers

**Figure 7:**
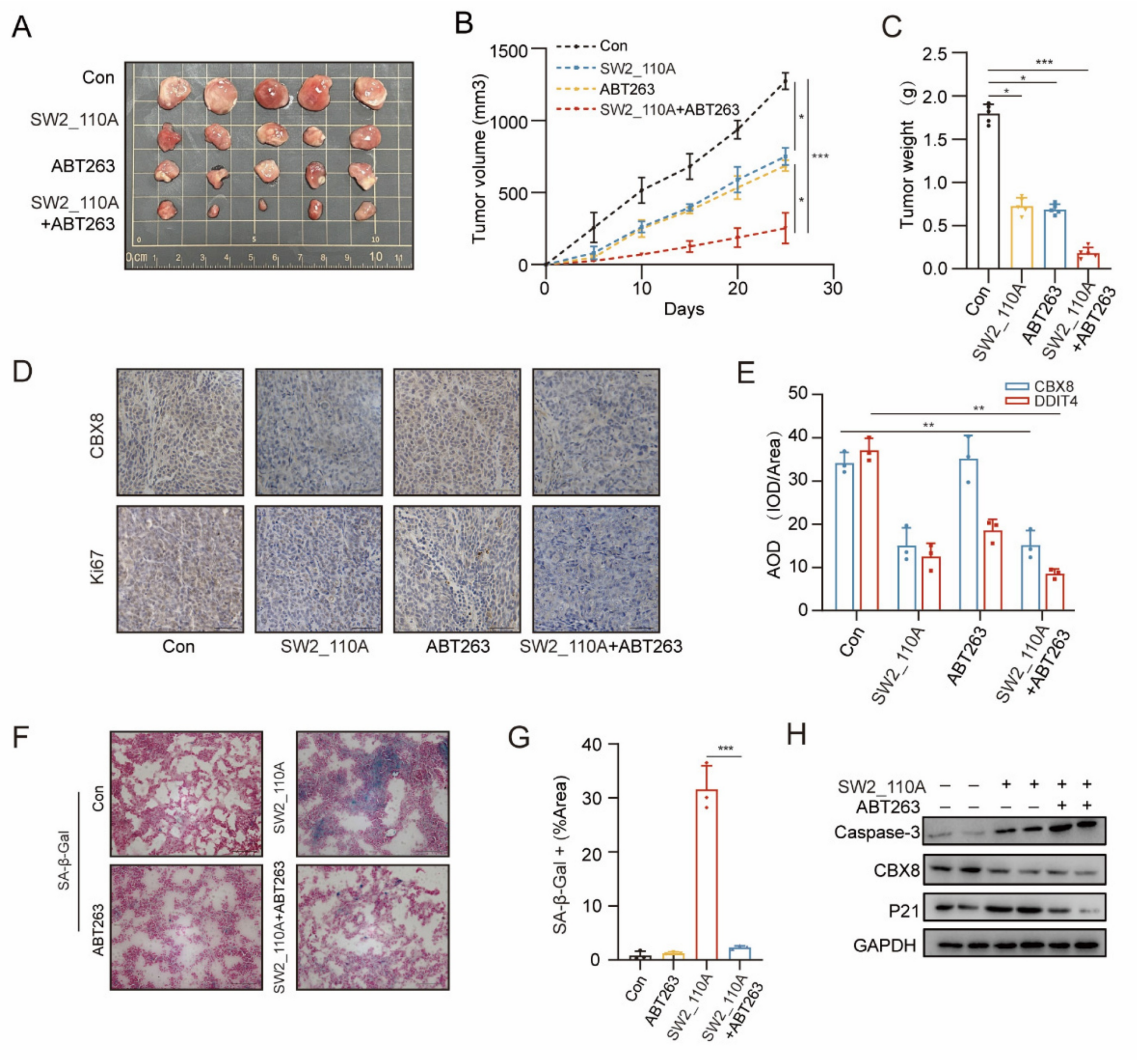
** Enhancement of antitumor effects in CRC by CBX8 inhibitors in combination with senescence scavengers. (A)** Representative images of xenograft tumors in nude mice after different treatments. SW2_110A and ABT263 were treated once a day for 25 days. The combination of SW2_110A with ABT263 had stronger inhibitory effects on tumor growth. **(B)** The tumor growth curves of each group of mice were summarized. **(C)** Tumor weights were examined in CDX as indicated. **(D-E)** IHC staining for Ki67 and CBX8 in the indicated groups was shown and AOD were quantified.** (F-G)** Representative images of SA-β-Gal staining of mouse tumors from the indicated groups, and the number of SA-β-Gal-positive cells in the indicated groups. **(H)** Western blot analysis of the indicated protein with SW2_110A and ABT263 treatment. The data represent the findings from three independent experiments and are shown as the means±SDs (*, *p*<0.05; **, *p*<0.01, ***, *p*<0.001). Scale bars: 50 μm (**d, f**).

ABT263 has been shown to efficiently eliminate various types of senescent cells, including senescent cancer cells, by reactivating the apoptotic pathway 22. To evaluate the therapeutic potential of combining CBX8 inhibition with ABT263, we established a xenograft mouse model by injecting HCT116 cells into nude mice, followed by treatment with senescence scavenger ABT263 (50 mg/kg) and a CBX8 inhibitor (25mg/kg). The combination of CBX8 inhibition and senescence scavengers exhibited synergistic antitumor effects, significantly reducing tumor growth and weight compared to either treatment alone (Figure 7A-C). Furthermore, IHC analysis confirmed that CBX8 expression was markedly suppressed by the CBX8 inhibitor, while Ki-67 levels were lower in the combination treatment group than in either monotherapy group, indicating enhanced suppression of tumor proliferation (Figure 7D-E). H&E staining of major mouse organs revealed no detectable histopathological changes following treatment (Figure S5A). To provide more comprehensive evidence of systemic safety, blood samples were collected for hematological and serum biochemical analyses, including WBC, RBC, PLT, ALT, AST, and creatinine. No significant alterations in any of these parameters were observed across all treatment groups compared with the control group (Figure S5B).

Xenograft tumors were cryosectioned, and SA-β-Gal activity was measured, confirming a significant reduction in tumor senescent cells in the combination therapy group compared to the SW2_110A group (Figure 7F-G). Western blot analysis revealed a significant upregulation of caspase-3 expression and a marked reduction in the senescence marker P21 in the combination therapy group (Figure 7H). Consistent with previous studies, these findings indicate that ABT263 effectively clears senescent cells by inducing apoptosis.

### Clinical relevance of CBX8 and DDIT4 in CRC

**Figure 8:**
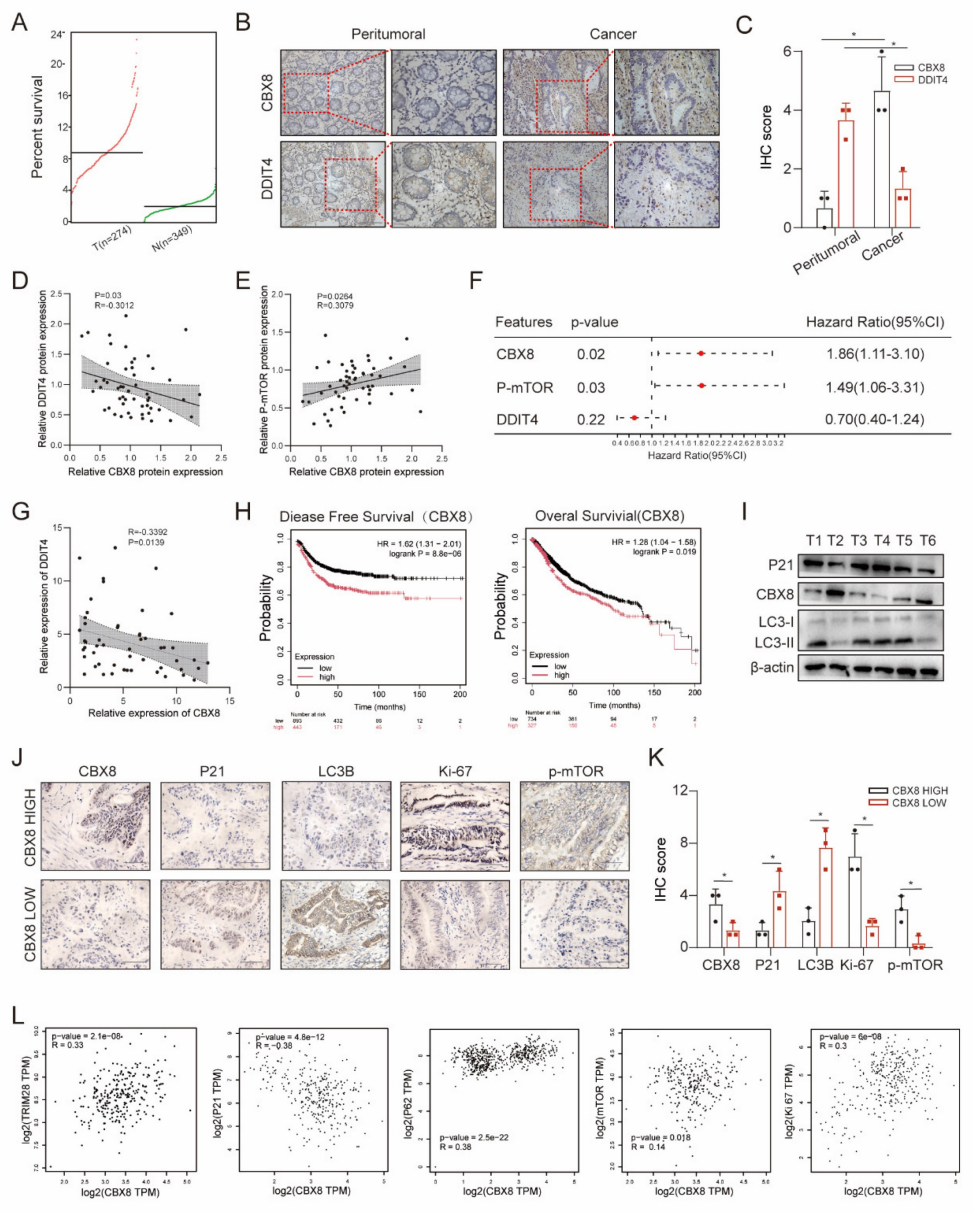
** Clinical relevance of CBX8 and DDIT4 in CRC. (A)** CBX8 expression was assessed using data from the TCGA.** (B-C)** The expression of CBX8 in peritumoral tissues and cancer tissues was assessed by IHC.** (D-E)** Association between CBX8 and DDIT4 protein expression in the clinical cohort.** (F)** Forest plot showing the results of multivariate Cox proportional hazards regression analysis for overall survival in the colorectal cancer cohort. Hazard ratios (HRs) and 95% confidence intervals (CIs) are shown for CBX8 and p-mTOR protein expression.** (G)** The correlation between CCBX8 and DDIT4 RNA expression obtained from qRT-PCR analysis. **(H)**The graph showed the results of Kaplan-Meier analysis of the disease-free survival (DFS) rate and the overall survival (OS) rate in CRC patients in the TCGA database with high or low expression of CBX8. **(I)** Expression of the indicated proteins was analyzed by immunoblotting in CRC specimens.** (J-K)** CBX8, P21, LC3B, Ki67, and p-mTOR expression, as assessed by IHC in patients, had relatively low and high levels of CBX8, and the IHC score was quantified. **(L)** The graph showed that CBX8 expression was correlated with TRIM28, P21, P62, mTOR and Ki67 expression in an analysis of TCGA data. The data represent the findings from three independent experiments and are shown as the means±SDs (*, *p*<0.05; **, *p*<0.01, ***, *p*<0.001). Scale bars: 50 μm (**b, j**).

Analysis of TCGA and GTEx data revealed that CBX8 expression was significantly elevated in colorectal cancer tissues compared to normal tissues (Figure 8A-C). Co-expression analyses performed in a cohort of 52 colorectal cancer patients revealed a significant inverse correlation between CBX8 and DDIT4 at the protein level, consistent with CBX8-mediated transcriptional repression of DDIT4 (Figure 8D). In contrast, CBX8 expression was positively correlated with p-mTOR protein levels, suggesting activation of the mTOR signaling pathway downstream of CBX8-mediated DDIT4 suppression (Figure 8E). Furthermore, multiple regression analysis showed that high CBX8 protein expression and high p-mTOR protein expression were identified as independent predictors of poor overall survival in our colorectal cancer cohort (Figure 8F). Consistently, similar trends were observed at the mRNA level (Figure 8G). Survival analyses of patients in the TCGA cohort further showed that high CBX8 expression was associated with shortened disease-free survival and overall survival (Figure 8H).

Next, we explored the relationship between CBX8 and cellular senescence or autophagy. Western blot analysis of colorectal cancer tissues showed that CBX8 expression was negatively correlated with P21 and LC3Ⅰ/Ⅱ in six colorectal cancer tissues (Figure 8I). Additionally, we further investigated the expression of key markers in tissues with high or low CBX8 expression by IHC. We observed that tumors with high CBX8 expression exhibited elevated levels of Ki-67 and p-mTOR, whereas the levels of P21 and LC3B were lower compared to tumors with low CBX8 expression (Figure 8J-K). Finally, dot plot analysis of TCGA data demonstrated significant correlations between CBX8 expression and multiple cellular markers, including TRIM28, P21, P62, mTOR, and Ki-67 (Figure 8L). These findings suggest a potential role for CBX8 in regulating senescence and autophagy in colorectal cancer.

## Discussion

Cellular senescence is characterized by irreversible cell cycle arrest coupled with extensive alterations in cellular morphology and metabolic activity 23. While most research on cellular senescence has concentrated on nonmalignant cells, emerging evidence indicates that neoplastic cells can also initiate a senescence response 24. However, the therapeutic vulnerabilities associated with senescence exhibit greater specificity. This concept underpins a sequential therapeutic strategy, known as a "one-two punch" approach, which induces tumor cell senescence followed by administration of a senolytic agent to eliminate these cells. This strategy represents a promising anticancer modality 22, 25. Furthermore, the mechanisms enabling neoplastic cells to evade senescence surveillance through epigenetic remodeling, metabolic reprogramming, or dynamic crosstalk with the tumor microenvironment during malignant progression remain inadequately defined 26-28. This study investigates the distinct contributions of cellular senescence to colorectal carcinogenesis and progression. We elucidate a novel mechanism wherein CBX8 acts as a transcriptional regulator that promotes oncogenesis through epigenetically mediated senescence modulation. We specifically demonstrate that the CBX8-TRIM28 complex orchestrates the spatiotemporal coordination of tumor cell autophagy and senescence through the DDIT4/mTOR signaling axis. Using experimental models, we further demonstrate that combining CBX8 targeting with senolytic therapy constitutes a novel "one-two punch" therapeutic strategy.

CBX8 constitutes a core component of the Polycomb CBX protein family 29. A specific subset of CBX proteins mediates recognition of chromatin architectural domains, which drives PRC1 complex assembly through targeted recruitment mechanisms 30. PRC1 recruitment is essential for maintaining target gene repression during differentiation and development 31. For example, CBX8, an essential component of the PRC1 complex, inhibits INK4a/ARF expression in fibroblasts 32. However, the role of CBX8 in modulating senescence-associated phenotypes within tumor cells remains inadequately investigated. Ablation of CBX8 induces profound transcriptional reprogramming that culminates in autophagy activation and subsequent senescence. This observation is consistent with emerging evidence positioning epigenetic regulators as critical arbiters of cell fate under stress conditions. Notably, our findings establish autophagy as a prerequisite for the ensuing senescence response in this context. Furthermore, CBX8 mediates evasion of senescence through an epigenetic-metabolic axis. The enrichment of H3K27me3, a canonical repressive histone mark, maintains condensed chromatin states and impedes the initiation of senescence programs 33. This further supports the concept that cellular senescence is not a passive process dictated solely by genetic sequence, but is dynamically regulated by a complex network of epigenetic modifications, including H3K27me3, and their regulatory factors 34.

The interplay between autophagy and senescence is highly dependent on cell type and context 35, 36. This is exemplified by studies demonstrating autophagy-promoted senescence in various scenarios: DNA damage-induced senescence 37, CDK inhibitor treatment in cancer 38, irradiation-induced senescence of cancer cells 6, and oncogene-induced senescence 39. Notably, the induction of cellular senescence also increases general autophagic flux, which may contribute to SASP secretion and cellular senescence status in the targeting of the rapamycin autophagy spatial coupling compartment (TASCC) 40. Our findings establish autophagy as a prerequisite for establishing the senescence phenotype in CBX8-deficient colorectal cancer cells. This likely originates from CBX8's context-specific regulation of metabolic stress: under DDIT4-mediated mTOR inhibition, autophagy facilitates SASP secretion through organelle clearance, rather than sustaining cell survival. This model is consistent with recent work demonstrating that mTOR suppression triggers senescence in an autophagy-dependent manner 18. However, it appears contradictory to studies in which autophagy inhibition promotes senescence 41. These apparent discrepancies may arise from variations in cell type, the intensity and duration of stress signals, or genetic background, highlighting the necessity for a context-dependent interpretation of the autophagy-senescence relationship.

DDIT4 operates as a critical stress-responsive metabolic checkpoint 42. Originally identified as a mediator of cellular stress responses, DDIT4 expression is upregulated under conditions including energy depletion, hypoxia, and DNA damage. Under these circumstances, it promotes energy conservation and damage repair by inhibiting mTORC1-driven anabolic processes 42-44. Although DDIT4 has established roles in metabolic adaptation and the energy stress response, its regulation by Polycomb proteins and functional contribution to senescence remained unexplored. Integrated ChIP-seq and transcriptomic analyses identified DDIT4 as a direct transcriptional target of CBX8. Subsequent functional rescue experiments demonstrated that DDIT4 knockdown abrogates senescence induced by CBX8 loss. These findings position the CBX8-DDIT4-mTOR axis as a central mechanism governing the autophagy-senescence transition. Notably, this study situates DDIT4 within an epigenetic regulatory framework by demonstrating that its basal expression is directly repressed by CBX8-dependent H3K27me3 marks in cancer cells. This constitutive repression effectively inactivates a crucial metabolic safeguard, thereby permitting sustained mTOR signaling and unabated proliferation even under basal conditions. Consequently, the epigenetic silencing of DDIT4 reveals a sophisticated mechanism whereby cancer cells rewire core stress response pathways to promote survival and growth. This positions DDIT4 not as a passive target, but as a pivotal node integrating epigenetic regulation with metabolic fate in CRC 45.

TRIM28 is conventionally characterized as a scaffolding protein that primarily exerts its repressive functions through H3K9me3-associated mechanisms 46, 47. By contrast, evidence implicating TRIM28 in the regulation of H3K27me3 remains relatively limited. Nevertheless, our observation that TRIM28 depletion leads to reduced H3K27me3 enrichment at the DDIT4 promoter expands the current understanding of the functional versatility of TRIM28. TRIM28 has been reported to physically associate with core PRC2 subunits, including EZH2, suggesting a potential capacity to stabilize local H3K27me3 deposition 48, 49. In addition, TRIM28 has been shown to influence histone methylation dynamics through the regulation of the H3K27-specific demethylase KDM6A 50. On this basis, it is plausible that TRIM28 regulates H3K27me3 through interactions with multiple chromatin-modifying proteins. Therefore, the specific complex and detailed mechanism of TRIM28 may need further exploration underlying this noncanonical role of TRIM28.

From a translational perspective, our findings position CBX8 as a promising therapeutic target. We demonstrate that CBX8 is overexpressed in CRC tissues and correlates with poor prognosis, consistent with its function as an oncogene that promotes escape from senescence. Notably, pharmacological inhibition of CBX8, when combined with the senolytic agent ABT263, synergistically inhibits tumor growth *in vivo* by eliminating senescent cells and activating apoptosis. This strategy aligns with increasing interest in senolytic therapies for cancer 51, 52, particularly in malignancies such as CRC that display high heterogeneity and resistance to conventional therapies 53. However, several challenges must be addressed in future studies, including optimization of treatment timing, patient stratification, and management of potential off-target effects. Additionally, it should be noted that cellular senescence in cancer represents a double-edged sword: while senescent cancer cells can activate anti-tumor immune surveillance, persistent senescent cells may also promote tumor heterogeneity and growth.

## Conclusion

In conclusion, we demonstrated that the CBX8/DDIT4/mTOR pathway is involved in regulating autophagy-dependent senescence in CRC cells. CBX8, a core component of the PRC1 complex, inhibits DDIT4 expression through H3K27me3-mediated epigenetic silencing. In addition, targeted inhibitors of CBX8 can be used as a "one-two punch" sequential treatment with senescence therapeutics. Our results suggest a therapeutic strategy for CRC targeting CBX8 to induce cellular senescence and then disrupt senescent cells.

## Supplementary Material

Supplementary methods, figures and tables.

## Data Availability

All data that support the findings are available from the corresponding author upon reasonable request.

## Abbreviations

AOM: Azoxymethane
AOD: Average Optical Density
ATG7: Autophagy Related 7
CBX8: Chromobox homolog 8
CCK-8: Cell counting kit-8
CDX: Cell line-derived xenograft
Co-IP: Co-immunoprecipitation
CRC: Colorectal cancer
CQ: Chloroquine
DDIT4: DNA-damage-inducible transcript 4
DFS: Disease-Free Survival
DSS: Dextran sulfate sodium
GSEA: Gene set enrichment analysis
HE: Hematoxylin and eosin
IB: Immunoblotting
IHC: Immunohistochemistry
IOD: Integrated Optical Density
KEGG: Kyoto Encyclopedia of Genes and Genomes
KO: Knockout
LC3B: Autophagy-Related Protein LC3 B
mTOR: Mammalian target of rapamycin
OS: Overall survival
PRC1: Polycomb repressive complex 1
P21(CDKN1A): Cyclin dependent kinase inhibitor 1A
P16(CDKN2A): Cyclin dependent kinase inhibitor 2A
P-S6: Phospho-S6 ribosomal protein
p-mTOR: Phospho-Mammalian target of rapamycin
RAPA: Rapamycin
S6: Ribosomal protein S6
shRNA: Short hairpin RNA
siRNA: Short interfering RNA
SASP: Senescence-associated secretory phenotypes
TEM: Transmission Electron Microscope
TRIM28: Tripartite motif containing 28
Vec: Vector
WB: Western blot
WT: Wildtype
