# Supplementary material for: CBX8 suppresses autophagy-dependent senescence in colorectal cancer by modulating the mTOR signaling pathway
Source: Int J Biol Sci. 2026 Feb 26;22(6):3127–43. doi: 10.7150/ijbs.126032 (PMC13050457; doi:10.7150/ijbs.126032)
Supplement: Supplementary file 1 — Supplementary methods, figures and tables. [file ijbsv22p3127s1.pdf]

Supplemental Methods

Reagents

The following antibodies were used: anti-CBX8 (Abcam, ab70796), anti-DDIT4 (Proteintech, 10638-1-AP), anti-TRIM28 (Proteintech, 15202-1-AP), anti-P16 (Proteintech, 10883-1-AP), anti-P21 (Proteintech, 10355-1-AP), anti-P62 (Proteintech, 18420-1-AP), anti-LC3B (CST, ab192890), anti-mTOR (Proteintech, 28273-1-AP), anti-GAPDH (CST, 2118), anti-β-actin (Proteintech, 20536-1-AP), anti-p-mTOR (Proteintech, 28879-1-AP), anti-Flag (CST, 2368), anti-Caspase-3 (Proteintech, 19677-1-AP), anti-S6 (Proteintech, 66886-1-Ig), anti-p-S6 (Proteintech, 29223-1-AP), anti-Ki67 (Proteintech, 27309-1-AP), anti-H3K27me3 (CST, 9733), anti-H3K9me3 (Abcam, ab8898), CQ (MCE, HY-17589A), RAPA (Sigma,553210), SW2_110A (MCE, HY-141716), AOM (MCE, HY-111375), DSS (MCE, HY-116282), and ABT263 (MCE, HY-10087), Fragments encoding CBX8 amino acid residues 1-60, 61-130, 131-240, 241-300, and 301-389 were generated by PCR and inserted into a pcDNA3.1-Flag vector, which was synthesized by GenePharma (Shanghai, China).

RNA isolation, real-time fluorescence quantitative PCR (RT-qPCR)

Total RNA was extracted using TRIzol Reagent (Invitrogen) and RNA purity was assessed using an ND-1000 Nanodrop. Each RNA sample had an A260:A280 ratio greater than 1.8 and an A260:A230 ratio greater than 2.0. Complementary DNA (cDNA) was generated using the PrimeScript RT kit (TaKaRa Bio) according to the manufacturer's instructions. The cDNA was used for qPCR on a CFX96 Real-Time Fluorescent Quantitative PCR Detection System (Bio-Rad) using SYBR Green PCR

premix (Life Technologies).

Western blot analysis

Cells or tissues were lysed in ice-cold radioimmunoprecipitation assay buffer containing protease inhibitors. After centrifugation at 12,000 rpm for 20 minutes at 4°C, lysates were separated by sodium dodecyl sulfate-polyacrylamide gel electrophoresis and transferred to polyvinylidene difluoride membranes for protein blotting using ECL detection reagent.

CCK-8 Assay

For proliferation assays, cells are inoculated in 96-well plates (2000 cells per well) and treated with the appropriate process. At the indicated time points, CCK8 was added to the wells and incubated with the cells according to the product instructions. Absorbance was measured at 450 nm and a growth curve was generated to determine the growth rate.

Colony formation assay

Cells were inoculated in 6-well plates (1000 cells per well), repeated 3 times, and then incubated in complete medium at 37°C and 5% CO_2 for 14 days. Cells were then fixed with methanol and stained with crystal violet and colonies were counted in each well.

Dual luciferase reporter assay

The promoter regions of CBX8 genes were PCR amplified and cloned into the pGL3 vector (Promega, Madison, WI). Cells were cotransfected with a firefly luciferase reporter expression constructs, a Renilla luciferase expression plasmid (pRL-TK, 20 ng) and shRNA(or overexpression plasmids). After 48 h, luciferase activity was measured

with a Dual Luciferase Reporter Assay System (Promega, Madison, WI).

In vivo tumor growth assay

Six-week-old male BALB/c nude mice (Shanghai Slac Laboratory Animal Co. Ltd., China) and C57BL/6J-Cbx8^{em1Cya} mice (Cyagen Biosciences, China) were obtainedand bred under specific pathogen-free conditions. CBX8-silenced cancer cells or control cells were subcutaneously injected into the flank regions of the mice (5 mice/group). Over a period of 5 weeks, tumor formation in the mice was observed by measuring the tumor volume. Then, the tumors were excised and weighed. All animal experiments were reviewed and approved by the Ethics Review Committee of Xuzhou Medical University.

Primers for qRT-PCR

Gene	Forward	Reverse
CBX8	ATACGGAAAGGACGCATGGAA	CTTGGGTCCACGCTTTTTGG
DDIT4	GATGCCTAGCCAGTTGGTAAG	CTAAACAGCCCCTGGATCTTG
CXCL10	CCAGAATCGAAGGCCATCAA	CATTTCCTTGCTAACTGCTTTCAG
ATG7	ATGATCCCTGTAACTTAGCCCA	CACGGAAGCAAACAACTTCAAC
TRIM28	CACUAGCUGUGAGGAUAAUTT	AUUAUCCUCACAGCUAGUGTT
IL-1α	AACCAGTGCTGCTGAAGGA	TTCTTAGTGCCGTGAGTTTCC
IL-1β	CTGTCCTGCGTGTTGAAAGA	TTGGGTAATTTTTGGGATCTACA
IL-6	CCAGGAGCCCAGCTATGAAC	CCCAGGGAGAAGGCAACTG
IL-8	CCCAGGGAGAAGGCAACTG	ATTGCATCTGGCAACCCTAC
IL-17	CTCTGTGATCTGGGAGGCAAA	CTCTTGCTGGATGGGGACA
IFN-β	AAACTCATGAGCAGTCTGCA	AGGAGATCTTCAGTTTCGGAGG
GAPDH	CTACCCACGGCAAGTTCAAC	CCAGTAGACTCCACGACATA

Primers for ChIP

Primer	Forward	Reverse
DDIT4 (-1790/-1650)	CACACTCCCGGGTCTGGA	TTGAATGGGTGTGCGAACC
DDIT4 (-1568/-1396)	CCTTGATCCTCCCCTGCC	CTCCATTGAGCCCCAGAAAG
DDIT4 (-1185/-1040)	GCCCTGGGCATCTGATGTAA	GCCATCCCGTGTTTCATCAT
DDIT4 (-923/-790)	GCCCTGGGCATCTGATGTAA	CAGCAGTGCTCAGAGGCTAACA
DDIT4 (-762/-622)	CCCAAATCAGCCTGGAGGA	CTTGGAGGGCTGGAGTAGATGA
DDIT4 (-437/-311)	GGCCCATCCACACTCCTCTA	GCCCATCCTGGCTGTTACTC
DDIT4 (-296/-169)	CCCAAATCAGCCTGGAGGAA	TGGATCAAGGCGGCTAGTTGT
DDIT4 (-92/88)	GGCTCCAACAAGCGACAGAG	TCATTTGACAGGTGGGAGACC

shRNA, siRNA and sgRNA sequence

CBX8 shRNA-1

CCGGCGTCACCATTAAGGAAAGTAACTCGAGTTACTTTCCTTAATGGTGAC GTTTTT

CBX8 shRNA-2

CCGGGCGTGAGCTTGGCATAGTGATCTCGAGATCACTATGCCAAGCTCACG CTTTTTG

CBX8 shRNA-3

CCGGACTTACGAGTTTCGAAGTGACCTCGAGGTCACTTCGAAACTCGTAA GTTTTTG

DDIT4 siRNA-1

ACACTTGTGTGCCAACCTG

DDIT4 siRNA-2

GCUGCUCAUUGAAGAGUGUTT

TRIM28 siRNA-1

CACTGAGGACTACAACCTT

TRIM28 siRNA-2

AGGACAGAGAACAGAGCCATT

ATG7 siRNA-1

UUCUCCGAACGUGUCACGUTT

ATG7 siRNA-2

GCUAGAGACGUGACACAUATT

TRIM28 sgRNA-1

GTTCGCATCCTGGGCGTCGG

TRIM28 sgRNA-2

AATTATTTCATGCGTGATAG
TRIM28 sgRNA-3
CTGGTTCGCATCCTGGGCGT

The luciferase reporter assay

The primers used for cloning the indicated promoters are as follows.

Gene	Forward	Reverse
DDIT4 (-1950/-780)	TGTGAACCCGGGAGGCGGAG	GTGCAGGGATGGAAGCGAGGAT
DDIT4 (-779/66)	GGCTAACGTCTGCTCCTGCG	GCCAACCACCGAGAGCCGCCCA

Supplementary Table 1 Candidate proteins were co-precipitated with CBX8

Gene names	Number of proteins	Sequence coverage [%]	Q-value	Score
SFPQ	7	36.8	0	304.7
NONO	9	43.7	0	247.44
ILF3	27	27.1	0	135.83
XRCC5	5	23.2	0	112.13
PTBP1	22	39.7	0	164.64
HNRNPL	21	28.4	0	150.06
SRSF1	11	33.6	0	80.783
HNRNPH1	26	41.7	0	236.19
EWSR1	16	17	0	118.88
BCLAF1	16	11.5	0	56.734
YBX1	7	50.3	0	146.47
THRAP3	1	12.1	0	74.976
TAF15	7	37.5	0	72.781
U2AF1	16	34.2	0	61.74
TMPO	1	23.3	0	110.9
TRIM28	5	11.4	0	56.479
SRSF9	5	25.8	0	46.108
DEK	9	18.1	0	39.396
FUBP1	10	10.5	0	38.391
PHB2	9	21.3	0	32.679
MCM5	11	9.1	0	30.315
PCBP1	17	25	0	52.669
HMGA1	6	48.6	0	42.412
REPIN1	8	7.6	0	24.266
U2AF2	4	8.9	0	21.769
CBX3	5	21.3	0	19.21
MCM7	10	6.9	0	16.852
KMT2B	5	6.2	0	31.989
TMPO	9	27.1	0	17.984
KHSRP	6	18.3	0	14.437
TRIM25	3	4.7	0	13.966
CCAR2	7	5.2	0	13.041
EEF1D	4	16.2	0	68.347
THRAP3	4	18.5	0	22.115
HMGA2	11	23.2	0	7.7783
DNMT1	6	10.1	0	7.6916
HMGA1	3	42.7	0	7.0219
CDC5L	3	1.9	0.0014749	6.499
EEF1D	26	29.9	0.0027435	6.0638
TP53	68	48.7	0.004065	5.9992

Supplementary Figures

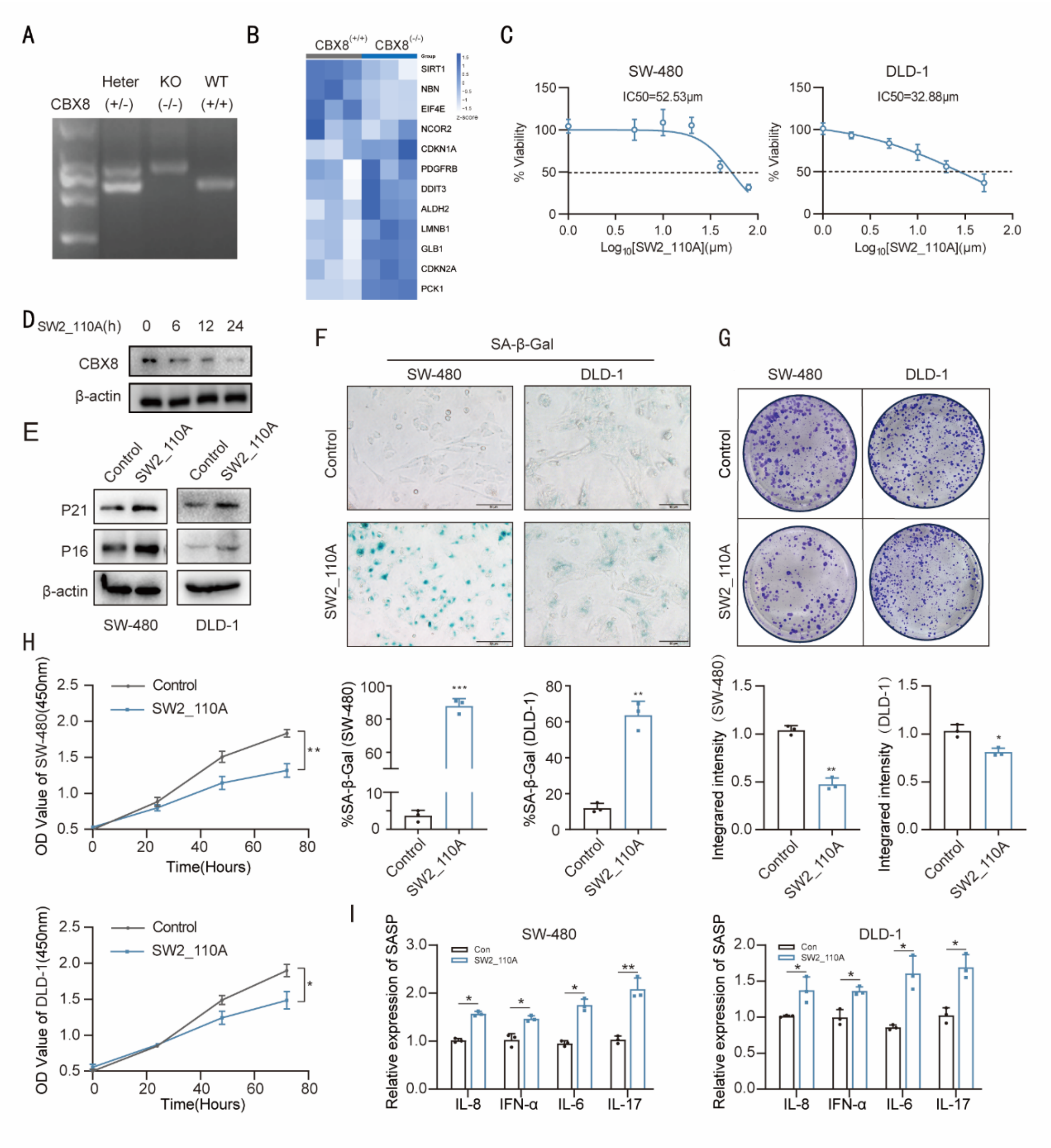

Supplementary Figure 1. Cellular senescence induction in CRC via CBX8 downregulation.

(A) Genomic DNA of CBX8 knockout（KO）mice analyzed by PCR. **(B)** Heatmap summarizing RNA-sequence results after CBX8 knockout for gene expression associated with the regulation of senescence. **(C)** DLD-1 and SW480 cells were treated with SW2_110A for 24 h. Cell viability was detected by CCK-8 assay and represented

as relative viability versus control. **(D)** Immunoblotting analysis of the indicated protein in SW2_110A treated at different time periods. **(E)** Immunoblotting analysis of the indicated protein with SW2_110A. **(F)** Representative images of SA-β-Gal staining of CRC cells with Control and SW2_110A treatment, and the number of SA-β-Gal-positive cells was quantified in the bottom panel. **(G)** Colony formation assays were performed on CRC cells to determine senescence-associated growth arrest and the integrated intensity for each group was quantified in the bottom panel. **(H)** Cell viability assays were performed on CRC cells to determine senescence-associated growth arrest. **(I)** Quantification of the abundance of the indicated mRNAs in CRC cells with SW2_110A treatment. The data represent the findings from three independent experiments and are shown as the means±SDs (*, p<0.05; **, p<0.01, ***, p<0.001). Scale bars: 100 μm (**f**).

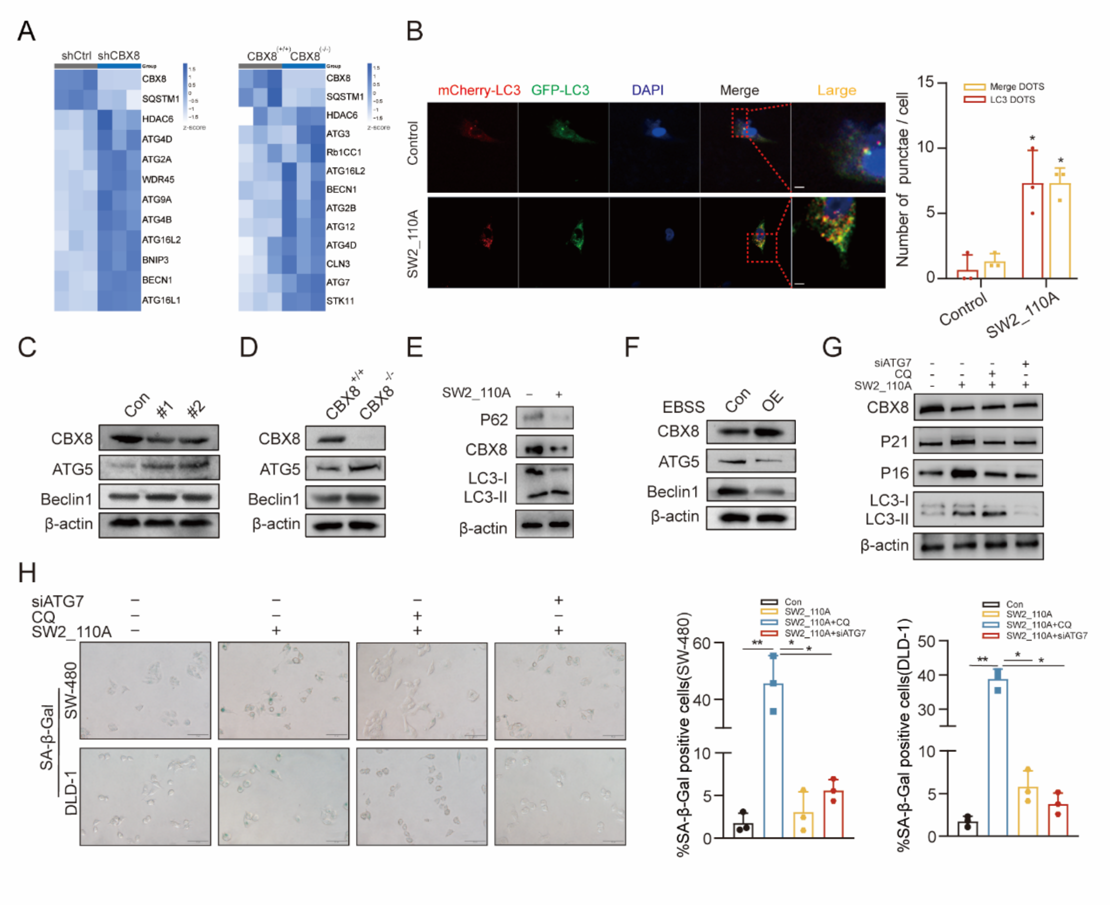

Supplementary Figure 2. CBX8 suppresses autophagy-dependent senescence in CRC cells.

(A) Heatmap summarizing RNA-sequence results after CBX8 knockdown and knockout for gene expression associated with the regulation of autophagy. **(B)** The effect of mCherry-GFP-LC3 point distribution in CBX8 inhibited cells with SW2_110A 48 hours treatment and CQ treatment for 24 hours after mCherry-GFP-LC3 plasmid transfection is shown by confocal laser scanning microscopy and the quantification of autophagic vesicles. **(C)** Immunoblotting of ATG5 and Beclin1 levels as indicated with shCBX8. **(D)** Immunoblotting of ATG5 and Beclin1 levels as indicated in the CBX8 knockout cells. **(E)** Immunoblotting of LC3 conversion, p62 levels as indicated with SW2_110A. **(F)** Immunoblotting of the indicated protein in the CBX8 OE cells. **(G)**

Immunoblotting analysis of the indicated protein with SW2_110A and siATG7 treatment. **(H)** SA-β-Gal activity staining was performed after SW2_110A treatment for 24 hours or siATG7 transfection, and the quantification of the percentage of SA-β-Gal-positive cells. The data represent the findings from three independent experiments and are shown as the means±SDs (*, *p*<0.05; **, *p*<0.01, ***, *p*<0.001). Scale bars: 10 μm (**b**), 100 μm (**h**).

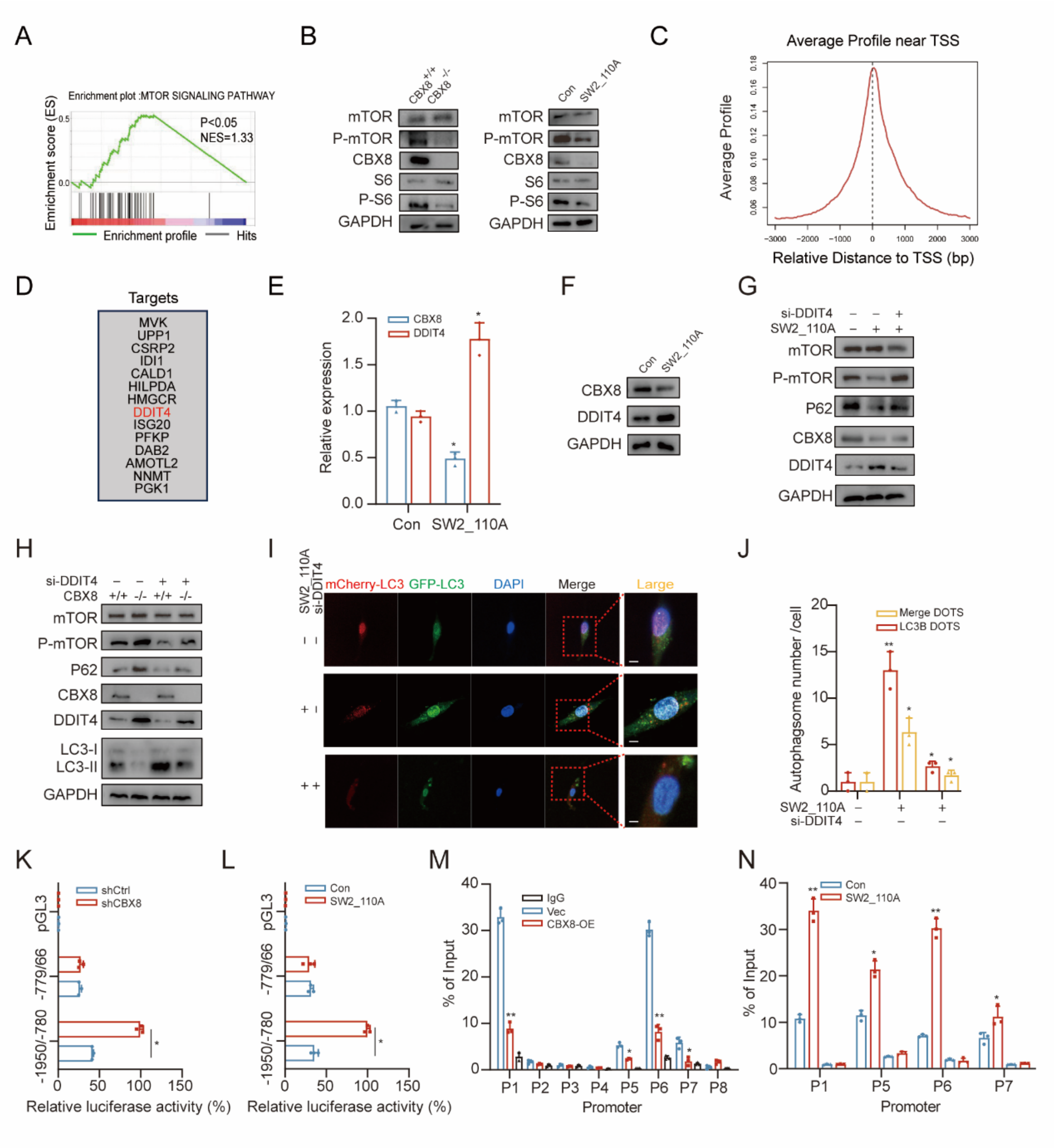

Supplementary Figure 3. CBX8 regulates autophagy-dependent senescence in

colorectal cancer through DDIT4-mediated modulation of mTOR signaling.

(A) GSEA analysis of transcriptome differential genes. **(B)** Immunoblotting of the indicated protein in cells of CBX8 WT and CBX8 KO and IB of the indicated protein with SW2_110A treatment. **(C)** CBX8 was preferentially distributed near the TSS of genes. **(D)** 14 genes overlapping in the Veen diagram. **(E)** The expression levels of DDIT4 were detected via qRT-PCR analysis. **(F)** IB analysis of the indicated protein with SW2_110A treatment. **(G)** IB showing the effects of CBX8 inhibition on the indicated protein levels in DLD-1 cells treated with siDDIT4. **(H)** IB showing the effects of CBX8 knockout on the indicated protein levels in cells treated with siDDIT4. **(I-J)** The effect of mCherry-GFP-LC3 point distribution in DLD-1 cells treated with SW2_110A and siDDIT4 24 hours after mCherry-GFP-LC3 plasmid transfection is shown by confocal laser scanning microscopy and the quantification of autophagic vesicles. **(K)** Luciferase reporter genes driven by the −1950/−780 or − 770/66 fragments of the DDIT4 promoter region were cotransfected with shCBX8 into DLD-1 cells, and luciferase activity was measured after 48 hours. The relative luciferase activity value in cells cotransfected with pRL-TK (−1950/−780) and SW2_110A was set to 100%. **(L)** Luciferase reporter genes driven by the −1950/−780 or − 770/66 fragments of the DDIT4 promoter region were cotransfected with SW2_110A into DLD-1 cells, and luciferase activity was measured after 48 hours. The relative luciferase activity value in cells cotransfected with pRL-TK (−1950/−780) and SW2_110A was set to 100%. **(M)** ChIP-qPCR analysis was used to determine the binding affinity of CBX8 to 8 DDIT4 promoter regions in SW480 cells. ChIP-qPCR

with IgG was performed as the control. **(N)** ChIP-qPCR analysis was used to determine the binding affinity of CBX8 to 8 DDIT4 promoter regions in DLD-1 cells. ChIP-qPCR with IgG was performed as the control. The data represent the findings from three independent experiments and are shown as the means±SDs (*, *p*<0.05; **, *p*<0.01, ***, *p*<0.001). Scale bars: 10 μm (**j**).

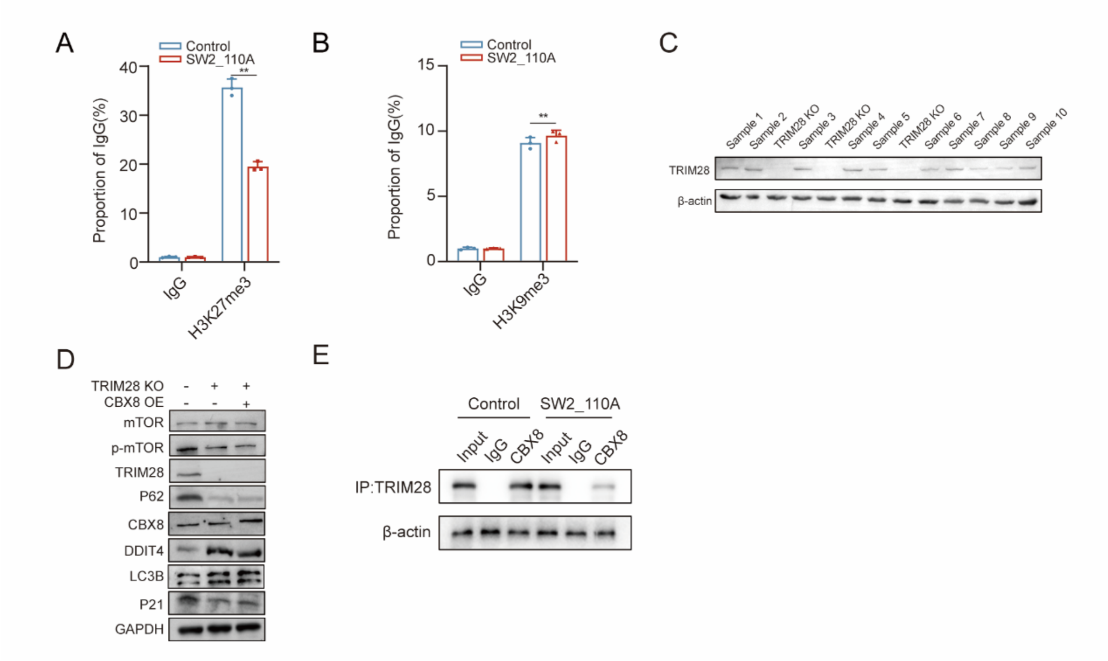

Supplementary Figure 4. CBX8 maintains the H3K27me3 status at the DDIT4 promoter by binding to TRIM28.

(A-B) ChIP-qPCR analysis identified enrichment of H3K27me3 and H3K9me3 after CBX8 suppression with SW2_110A in DLD-1 cells. ChIP-qPCR with IgG was performed as the control. **(C)** Immunoblotting of TRIM28 expression in SW480 cells. **(D)** Immunoblotting of the indicated protein in TRIM28 knockout in SW480 cells overexpressing CBX8. **(E)** CoIP showed the interaction between the CBX8 and TRIM28 proteins in DLD-1 cells with SW2_110A. The data represent the findings from three independent experiments and are shown as the means±SDs (*, *p*<0.05; **,

p<0.01).

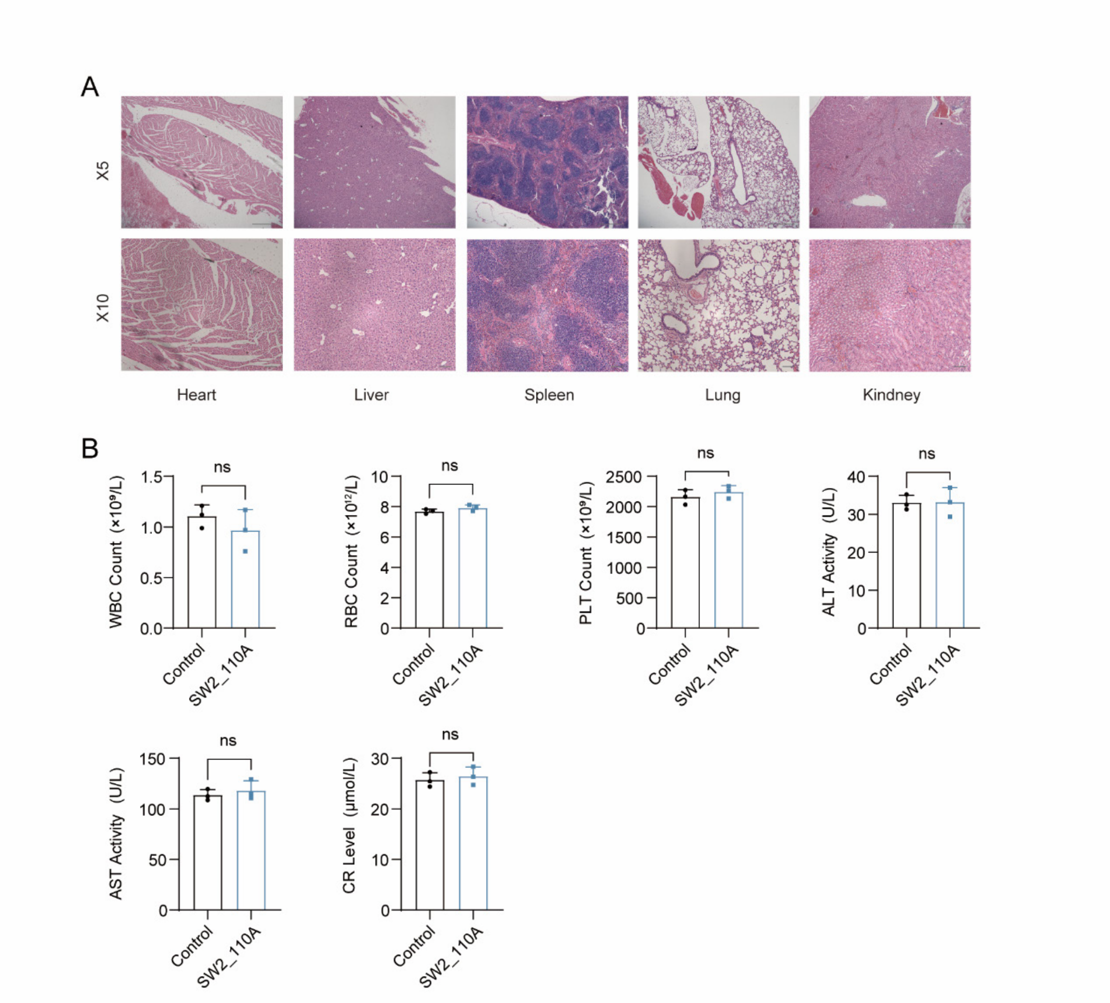

Supplementary figure 5. Assessment of the in vivo systemic safety of the CBX8-targeting compound SW2_110A.

(A) Staining plots of various organs of CBX8 knockout mice after application of SW2_110A. **(B)** Analysis of systemic toxicity based on serum biochemistry: alanine aminotransferase (ALT), aspartate aminotransferase (AST), creatinine (CR) and complete blood count: white blood cells (WBC), red blood cells (RBC), platelets (PLT) confirms all measured parameters were within normal physiological limits across all treatment groups. Scale bars: 100 μm.
